# Caffeoylquinic Acids From *Lonicera japonica* Thunb. as Hypoglycemic Agents: Network Pharmacology and Pharmacological Validation

**DOI:** 10.1155/jdr/6712215

**Published:** 2026-05-30

**Authors:** Yiyi Ye, Dawei Xu, Shilu Zhang, Huawei Yu, Yingwei Liu, Jin Yang, Lijuan Sun

**Affiliations:** ^1^ Institute of Chinese Traditional Surgery, LongHua Hospital, Shanghai University of Traditional Chinese Medicine, Shanghai, Shanghai, China, shutcm.edu.cn; ^2^ Hubei Province Key Laboratory of Biotechnology of Chinese Traditional Medicine, School of Health Science and Engineering, Hubei University, Wuhan, Hubei, China, hubu.edu.cn; ^3^ Medical School, Hubei Minzu University, Enshi, Hubei, China, hbmy.edu.cn

**Keywords:** caffeoylquinic acids, gluconeogenesis, hypoglycemic activity, *Lonicera japonica* Thunb., Type 2 diabetes

## Abstract

The global increase in Type 2 diabetes (T2D) presents a serious public health challenge. *Lonicera japonica* Thunb., a traditional medicinal and edible plant, is a rich source of caffeoylquinic acids (CQAs), which are naturally occurring polyphenols that have drawn considerable research interest due to their diverse biological activities, particularly their potential in the management of T2D. In this study, six CQAs were isolated from the flower buds of *L. japonica* using preparative HPLC, with a total yield of 0.0555 mg/g. These compounds were identified as 3‐CQA, 4‐CQA, 5‐CQA, 3,4‐DCQA, 3,5‐DCQA, and 4,5‐DCQA, and their structures were characterized by LC‐DAD‐ESI‐QTOF‐MS and NMR. Hypoglycemic activity was then assessed using *α*‐glucosidase/*α*‐amylase inhibition, glucose uptake, and hepatic glucose production assays. Network pharmacology predicted potential targets and pathways, which were then validated by dual‐luciferase reporter and Western blot analyses. Results showed that CQAs selectively inhibited *α*‐glucosidase (not *α*‐amylase), enhanced glucose uptake, and suppressed hepatic glucose production. Among them, the di‐CQAs, particularly 4,5‐DCQA and 3,5‐DCQA, were the most potent *α*‐glucosidase inhibitors, with IC₅₀ values of 5.49 and 7.96 mM, respectively. Network analysis identified AKT1, PEPCK, and INSR as core targets, and PI3K/AKT, insulin, and FoxO signaling pathways as key pathways. Mechanistically, 3,5‐DCQA suppressed gluconeogenesis by inhibiting PEPCK (at both promoter activity and protein levels) and downregulating CREB. It also enhanced insulin signaling via upregulation of INSR, IRS‐1, and p‐AKT. In conclusion, our study demonstrates that 3,5‐DCQA, a key bioactive constituent of *L. japonica*, exerts multitarget hypoglycemic effects by coordinately regulating gluconeogenesis and insulin signaling. These findings not only provide a direct pharmacological basis for the antidiabetic potential of CQA‐rich herbs but also highlight 3,5‐DCQA as a promising candidate for developing novel therapeutics or adjuncts for T2D management.

## 1. Introduction

Diabetes is a metabolic disorder characterized by chronic hyperglycemia due to defective insulin secretion or action. It is classified into Type 1 diabetes (T1D), resulting from autoimmune *β*‐cell destruction; Type 2 diabetes (T2D), characterized by insulin resistance and progressive *β*‐cell dysfunction; and other specific types [1]. T2D, the most common form, represents the majority of cases worldwide and poses a growing public health challenge due to its rising prevalence and complications. The pathogenesis of T2D involves genetic susceptibility and environmental factors such as obesity, sedentary behavior, and aging [2], which contribute to *β*‐cell failure and impaired insulin signaling in skeletal muscle, liver, and adipose tissue, ultimately disrupting glucose homeostasis.

The global rise in diabetes and growing demand for natural health products underscore the need for safer therapies. Current T2D management relies on synthetic hypoglycemic agents, including insulin secretagogues, sensitizers, *α*‐glucosidase inhibitors, as well as newer classes such as SGLT2 inhibitors and GLP‐1 receptor agonists [3]. Despite their efficacy, these drugs often cause adverse effects, driving the search for plant‐derived alternatives. Traditional food and medicinal plants offer promising sources of hypoglycemic agents with improved safety profiles and multitarget mechanisms for sustainable glycemic control.

*Lonicera japonica* Thunb., a medicinal and edible herb, has a long history of use in China both for treating conditions such as the common cold, sore throat, and skin infections, and as a tea, as well as in Korean medicine for managing diabetes and obesity [4, 5]. This herb is a rich source of caffeoylquinic acids (CQAs), which are shikimate pathway‐derived phenylpropanoids [6] that are also abundant in *Eucommia ulmoides* and *Coffea* species. Pharmacological studies have demonstrated that CQAs exhibit diverse bioactivities, including antioxidant [7–9], anti‐inflammatory [10–13], and metabolic regulatory effects (e.g., hypoglycemic [8, 14] and lipid lowering [13, 15]). They also have shown organoprotective effects such as hepatoprotective [16, 17], cardioprotective [18], and neuroprotective activities [19], along with antimicrobial actions (bactericidal [7] and antiviral [20] effects) and therapeutic potential (antitumor [21] and antihypertensive [22] properties). These multifaceted bioactivities highlight CQAs as a key focus in modern phytochemical research.

**Figure 1:**
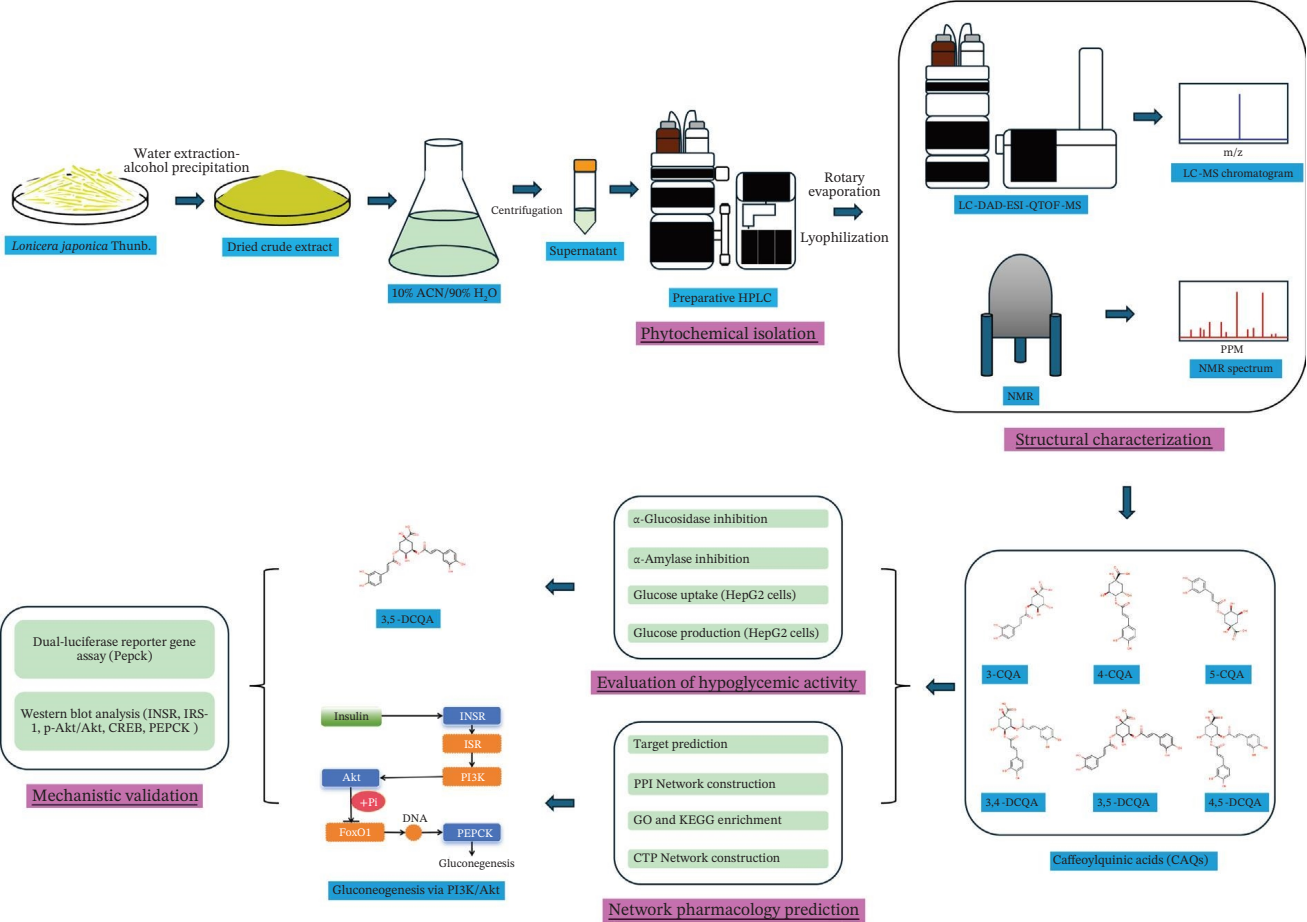
Schematic workflow for the discovery and mechanistic validation of the antidiabetic component from *L. japonica.*

CQAs protect pancreatic *β*‐cells by suppressing apoptosis and enhancing insulin secretion, thereby improving glycemic control and underscoring their therapeutic potential for T2D [23]. However, the reliable analysis and unambiguous characterization of individual CQAs remain technically challenging, largely due to the presence of numerous isomers with similar physicochemical properties [6]. This limitation has consequently hindered systematic comparisons of the antidiabetic activities of high‐purity CQA compounds from a defined botanical source, thereby impeding a clear understanding of their structure‐activity relationships. In this study, CQA compounds were isolated from *L. japonica*, purified, structurally characterized, and systematically evaluated for their antidiabetic activities in vitro. Network pharmacology was then employed to predict their potential targets, followed by experimental validation. This integrated strategy is summarized in Figure 1. The present study is aimed at providing a clearer phytochemical basis for the traditional use of *L. japonica* and to explore the structure‐activity relationships of CQAs against T2D.

## 2. Methods and Materials

### 2.1. Reagents and Chemicals

Acetonitrile, acetic acid, sodium carbonate, and hydrochloric acid were obtained from Sinopharm Group Chemical Reagent Co., Ltd. (Shanghai, China). Dimethyl sulfoxide (DMSO), 3‐(4,5‐dimethylthiazol‐2‐yl)‐2,5‐diphenyl‐2H‐tetrazolium bromide (MTT), glutathione, *p*‐nitrophenyl‐*α*‐D‐glucopyranoside (*p*NPG), acarbose, glucose assay kit, 2‐[N‐(7‐nitrobenz‐2‐oxa‐1,3‐diazol‐4‐yl) amino]‐2‐deoxy‐D‐glucose (2‐NBDG), metformin, iodine, RIPA lysis buffer, phenylmethanesulfonyl fluoride (PMSF), protease inhibitor cocktail, sodium dodecyl sulfate (SDS), and polyacrylamide were sourced from Aladdin Scientific (Shanghai, China). Dulbecco′s Modified Eagle′s Medium (DMEM), penicillin‐streptomycin, *α*‐glucosidase, *α*‐amylase, insulin, Tween‐20, and the enhanced chemiluminescence (ECL) kit were purchased from Servicebio Technology Co., Ltd. (Wuhan, China). The Pierce BCA protein assay kit was procured from Thermo Fisher Scientific (Waltham, Massachusetts, United States). The dual‐luciferase reporter kit and FuGENE were obtained from Promega Corporation (Madison, Wisconsin, United States). Fetal bovine serum (FBS) was sourced from Life Technologies Corporation (Carlsbad, California, United States). Antibodies against insulin receptor (INSR), insulin receptor substrate 1 (IRS‐1), p‐AKT, AKT, cAMP response element‐binding protein (CREB), phosphoenolpyruvate carboxykinase (PEPCK), and *β*‐actin were purchased from Cell Signaling Technology, Inc. (Danvers, Massachusetts, United States).

### 2.2. Extract Preparation and Compound Isolation

Flower buds of *L. japonica* were collected in Fengqiu County, Henan Province, China, in September 2021. The materials were identified by Prof. Hezhen Wu from Hubei University of Traditional Chinese Medicine, with a voucher specimen (PA20210012) deposited at the Hubei Province Key Laboratory of Biotechnology of Chinese Traditional Medicine. The extraction of CQAs from *L. japonica* flower buds was performed using a hot water extraction method [24, 25]. For extraction, 1 kg of raw sample was subjected to three successive extractions using 10‐L boiling water per extraction. The combined aqueous extracts were concentrated to a relative density of 1.20 g/mL. The concentrate underwent precipitation with four volumes of 95% ethanol (*v*/*v*). Following centrifugation, the supernatant was concentrated to complete dryness. The dried crude extract was redissolved in 10% (*v*/*v*) acetonitrile aqueous solution with sonication‐assisted complete solubilization. After centrifugation, the supernatant was subjected to preparative HPLC separation under the following conditions: Agilent ACE HL‐C18 column (10 × 150 mm, 5 *μ*m); mobile phase of 0.1% (*v*/*v*) acetic acid (A) and acetonitrile (B) at a flow rate of 4 mL/min; column temperature of 25°C; 45‐min gradient elution from 15% to 25% B, then a 0.5‐min ramp to 95% B; detection at 324 nm (200–800 nm scanning range).

Eluates were collected based on retention time, concentrated via rotary evaporation under reduced pressure, and lyophilized. For structural characterization, aliquots were analyzed by LC‐DAD‐ESI‐QTOF‐MS and further confirmed by NMR spectroscopy.

### 2.3. LC‐DAD‐ESI‐QTOF‐MS Analysis

The chemical composition of the sample was profiled using LC‐DAD‐ESI‐QTOF‐MS, with chromatographic and MS acquisition parameters set as previously reported [26]. Separation was performed on an Agilent Zorbax SB‐C18 column (150 × 4.6 mm, 5 *μ*m) maintained at 35°C. The mobile phase, delivered at 1.0 mL/min, consisted of 0.1% (*v*/*v*) formic acid in water (Solvent A) and acetonitrile (Solvent B). A linear gradient was programmed as follows: 5%–15% B from 0 to 15 min, then 15%–40% B from 15 to 40 min. The DAD detection wavelength was set at 280 nm. After the column, the flow was split using a T‐splitter, with approximately 1% of the effluent directed to the MS. Data processing and analysis, including mass axis calibration, chemical formula prediction, and theoretical mass calculation, were performed using Bruker DataAnalysis 4.0 software.

### 2.4. NMR Experiment

NMR analysis was performed according to a reported method [26]. All ^1^H NMR spectra were acquired at 600 MHz using a Bruker Avance III spectrometer equipped with a 5 mm cryogenic TXI probe, with data acquisition performed using the Topspin 3.5.7 software. The zg pulse sequence was applied with the following acquisition parameters: 32 transients, 32 k data points, a spectral width of 20 ppm, and a relaxation delay of 2 s. Chemical shifts (*δ*_H_) were referenced to the residual solvent signal of CD₃OD, using a reference value of *δ*_H_ 3.21.

### 2.5. Target Prediction for CQAs

The CAS numbers and 3D structures of the isolated CQAs were retrieved from SciFinder [27] and PubChem (https://pubchem.ncbi.nlm.nih.gov) [28]. Compound structures were generated using Chem3D, exported in SDF format, and imported into SwissTargetPrediction (https://www.swisstargetprediction.ch/) [29] and PharmMapper (https://lilab-ecust.cn/pharmmapper/index.html) [30] for target prediction, with species restricted to *Homo sapiens*. Targets with nonzero probability were screened, and duplicates were removed based on the prediction results. The remaining targets were further validated using the UniProt database (https://www.uniprot.org/) [31] to obtain the final target list for CQA compounds.

### 2.6. Acquisition of Hyperglycemia‐ and T2D‐Related Targets

The keywords “hyperglycemia,” “T2DM,” “Type II diabetes mellitus,” and “Type 2 diabetes mellitus” were used to search for genes associated with abnormal glucose metabolism in *H. sapiens*. The GeneCards database (https://www.genecards.org/) [32] and OMIM database (https://omim.org/search/advanced/geneMap) [33] were employed, with a relevance score > 3 as the screening criterion. The resulting gene targets were validated and deduplicated using the UniProt database (https://www.uniprot.org/) to generate a final list of hyperglycemia‐related genes.

### 2.7. Identification of Common Targets Between CQAs and Diabetes

The potential therapeutic targets of CQAs for blood glucose regulation were identified by intersecting the predicted compound targets with hyperglycemia‐related targets using the Venny 2.1.0 platform (https://bioinfogp.cnb.csic.es/tools/venny/index.html).

### 2.8. Construction of Protein–Protein Interaction (PPI) Network

The targets were imported into the STRING database (https://string-db.org/) [34] to generate a PPI network for *H. sapiens* with a confidence score > 0.9. Unconnected nodes were removed, and the network was visualized and analyzed using Cytoscape 3.9.1 [35] to construct the final PPI network diagram.

### 2.9. Enrichment Analysis

The potential therapeutic targets of CQAs were analyzed through Gene Ontology (GO) functional enrichment and Kyoto Encyclopedia of Genes and Genomes (KEGG) pathway analysis using the DAVID 2021 database (https://david.ncifcrf.gov/) [36]. Results were filtered based on the enrichment scores, and visualization was performed using the bioinformatics platform (https://www.bioinformatics.com.cn/) to generate GO bar plots and KEGG bubble diagrams.

### 2.10. Topology Network Construction

Based on the enrichment results, pathways related to blood glucose regulation, their associated targets, and CQAs were imported into Cytoscape 3.9.1 for topology analysis and network visualization. The resulting “compound‐target‐pathway” network illustrated the complex interactions among these components.

### 2.11. Cell Line and Culture Conditions

The HepG2 human hepatoma cell line, obtained from the State Key Laboratory of Virology (Wuhan University, China), was cultured in DMEM supplemented with 10% FBS and 1% penicillin‐streptomycin at 37°C in a humidified 5% CO_2_ atmosphere.

### 2.12. Cell Viability Assay

Cell viability was assessed using a standard MTT assay [37]. HepG2 cells in the logarithmic growth phase were seeded in 96‐well plates at a density of 2 × 10^5^ cells/well. Following a 24‐h incubation, cells were treated with varying concentrations of CQAs (50–1000 *μ*M) for an additional 24 h. The medium was then replaced with 200 *μ*L of MTT solution (0.5 mg/mL) and incubated at 37°C in the dark for 4 h. After complete removal of the MTT solution, formazan crystals were dissolved in 100 *μ*L of DMSO per well. Absorbance was measured at 570 nm, with cell viability calculated as a percentage of untreated controls (set as 100%).

### 2.13. *α*‐Glucosidase Inhibition Assay

*α*‐Glucosidase inhibition was assessed using a modified pNPG‐based method [38]. The enzymatic reactions were performed in 96‐well plates containing 50 *μ*L of 0.2 M phosphate buffer (pH 6.8), 40 *μ*L of CQAs (2, 6, or 10 mM), 40 *μ*L of *α*‐glucosidase (0.5 U/mL), and 10 *μ*L of glutathione (0.2 U/mL). Following a 30‐min incubation at 37°C, 45 *μ*L of 5.0 mM pNPG was added and incubated for an additional 30 min. Reactions were terminated with 50 *μ*L of 0.5 M sodium carbonate, with acarbose as the positive control. Absorbance at 405 nm was measured using a Berthold TriStar LB941 microplate reader (Bad Wildbad, Germany), and IC₅₀ values were determined using Origin 8.6 software.

### 2.14. *α*‐Amylase Inhibition Assay

*α*‐Amylase inhibition was assessed by a modified starch‐iodine method [39]. A reaction mixture containing 35 *μ*L of *α*‐amylase (1 mg/mL) and 35 *μ*L of CQAs (2.5, 5, or 10 mM) was incubated in a 96‐well plate at 37°C for 30 min. Subsequently, 80 *μ*L of 0.1% starch solution was added, followed by an additional 30‐min incubation. The reaction was terminated with 100 *μ*L of 0.1 M HCl, and 60 *μ*L of iodine solution was added. Absorbance was measured at 590 nm using a microplate reader, with acarbose as the positive control. IC₅₀ values were calculated using Origin 8.6 software.

### 2.15. 2‐NBDG Uptake Assay

HepG2 cells were seeded in 12‐well plates at a density of 2 × 105 cells/well and cultured for 24 h. The cells were then treated with CQAs (25, 50, or 100 *μ*M) for an additional 24 h. After removing the medium, cells were washed three times with ice‐cold PBS. Glucose starvation was performed using glucose‐free medium for 4 h, followed by incubation with 50 *μ*M 2‐NBDG for 30 min. Fluorescence intensity (excitation/emission: 485/535 nm) was measured using a microplate reader. Protein concentrations were determined by the Pierce BCA assay for data normalization.

### 2.16. Hepatocyte Glucose Production Assay

HepG2 cells were seeded in 6‐well plates at 2 × 10^5^ cells/well and cultured for 24 h, followed by serum starvation for 12 h. Cells were treated with CQAs (25, 50, or 100 *μ*M) for 24 h, washed three times with PBS, and incubated in glucose production buffer (containing glucose‐ and phenol red‐free DMEM with 2 mM sodium pyruvate and 20 mM sodium lactate) for 4 h. Medium aliquots (30 *μ*L) were collected for glucose measurement using a glucose assay kit, with insulin as the positive control. Data were normalized to cellular protein content.

### 2.17. Dual‐Luciferase Reporter Gene Assay

HepG2 cells were seeded in 24‐well plates and cultured until reaching 60%–70% confluence (24 h). Cells were cotransfected with pGL3‐PEPCK‐promoter and pRL‐TK plasmids using FuGENE according to the manufacturer′s protocol. After 24 h, cells were treated with CQAs (25, 50, or 100 *μ*M) for 24 h. Following ice‐cold PBS washing, cell lysates were prepared for dual‐luciferase activity measurement. Firefly luciferase activity was normalized to Renilla luciferase activity in each sample, with metformin as the positive control.

### 2.18. Western Blot Analysis

HepG2 cells cultured in 6‐well plates were treated with 3,5‐DCQA (25, 50, or 100 *μ*M) for 24 h. Cells were lysed in RIPA buffer supplemented with protease and phosphatase inhibitors (1 mL RIPA buffer containing 10 *μ*L of 100× PMSF, 25 *μ*L of 40× phosphatase inhibitor, and 10 *μ*L of 100× protease inhibitor cocktail). After centrifugation, protein concentrations were quantified using the Pierce BCA assay.

For Western blot analysis, protein lysates (30 *μ*g per sample) were separated by 10% SDS‐PAGE and transferred to a nitrocellulose membrane. The membranes were blocked with 5% skimmed milk for 1 h, followed by overnight incubation at 4°C with primary antibodies (1:1000 dilution). After washing with TBST (0.05% Tween‐20), the blots were incubated with secondary antibodies (1:1000) for 1 h at room temperature. Protein bands were visualized using an ECL kit, and chemiluminescence was captured with a FluorChem imaging system (Alpha Innotech, San Leandro, California, United States). Band intensities were quantified using Multi‐Gauge V3.1 software (FUJIFILM Corporation, Tokyo, Japan), with *β*‐actin as the loading control.

### 2.19. Statistical Analysis

Statistical analyses were performed using one‐way ANOVA followed by Tukey′s post hoc test in GraphPad Prism 8.0 software. Quantitative data are expressed as mean ± standard deviation (SD) from three independent experiments. A *p* value < 0.05 was considered statistically significant.

## 3. Results and Discussion

### 3.1. Extraction, Isolation, and Identification of CQAs

Although both hot‐water and ethanolic extraction methods are standard for isolating CQAs from *L. japonica* [24, 25, 40, 41], this study employed hot‐water extraction due to its alignment with traditional decoction practices and its reported comparable yields of key CQAs (e.g., 5‐CQA) to those of ethanolic extraction [42]. Following this, ethanol was used solely to precipitate impurities such as polysaccharides and proteins. This approach minimized solvent use, cost, and safety risks, and yielded an extract suitable for subsequent separation and bioassays.

**Figure 2:**
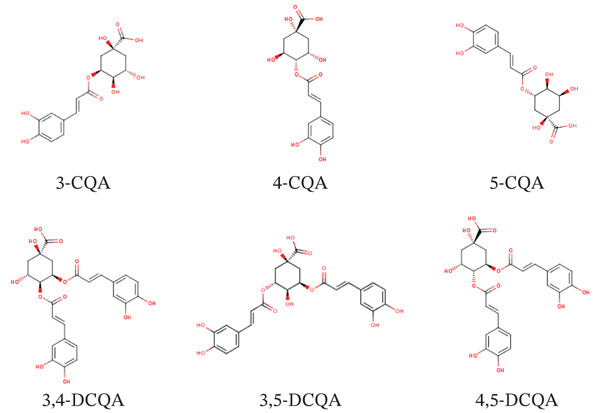
Chemical structures of six CQAs isolated from *L. japonica*. The compounds were purified by preparative HPLC and their structures were elucidated by LC‐DAD‐ESI‐QTOF‐MS and NMR analyses. They include three mono‐CQAs (3‐CQA, 4‐CQA, and 5‐CQA) and three di‐CQAs (3,4‐DCQA, 3,5‐DCQA, and 4,5‐DCQA).

From this extract, six CQA compounds from *L. japonica* were unequivocally identified following isolation by preparative HPLC as 3‐CQA, 4‐CQA, 5‐CQA, 3,4‐DCQA, 3,5‐DCQA, and 4,5‐DCQA (Figure 2). Their structural identification was supported by comprehensive analysis of LC‐DAD‐ESI‐QTOF‐MS and NMR data (Figures S1–S6). The total isolated yield was 55.5 mg (0.0555 mg/g plant material), with all compounds exceeding 95% purity. The individual yields are detailed in Table 1.

**Table 1:** Yields and purities of CQAs isolated from *L. japonica*.

No.	Compound	Yield (mg)	Yield (mg/g plant material)	Purity (%)
C1	3‐O‐caffeoylquinic acid (3‐CQA)	2.05	0.0021	> 95%
C2	4‐O‐caffeoylquinic acid (4‐CQA)	7.80	0.0078	> 95%
C3	5‐O‐caffeoylquinic acid (5‐CQA)	6.20	0.0062	> 95%
C4	3,4‐di‐O‐caffeoylquinic acid (3,4‐DCQA)	11.75	0.0118	> 95%
C5	3,5‐di‐O‐caffeoylquinic acid (3,5‐DCQA)	10.49	0.0105	> 95%
C6	4,5‐di‐O‐caffeoylquinic acid (4,5‐DCQA)	17.21	0.0172	> 95%
	Total	55.50	0.0555	

#### 3.1.1. Identification of 5‐CQA

The quasi‐molecular ion at *m*/*z* 353.0770 and a key fragment at *m*/*z* 191.0556 observed in the MS spectrum were consistent with 5‐CQA [26]. In the ^1^H NMR spectrum, a pair of coupled protons at *δ* 7.57 (d, *J* = 16.0 Hz) and 6.27 (d, *J* = 16.0 Hz), along with three aromatic protons at *δ* 7.06 (d, *J* = 2.0 Hz), 6.97 (dd, *J* = 8.2, 2.0 Hz), and 6.79 (d, *J* = 8.2 Hz), confirmed the caffeoyl moiety. Additionally, the characteristic splitting pattern of the proton at *δ* 5.34 (ddd, J = 11.2, 9.5, 4.8 Hz) was assigned to H‐5 of the quinic acid residue, further verifying the structure.

#### 3.1.2. Identification of 3‐CQA

The quasi‐molecular ion at *m*/*z* 353.0770 and key fragment ions at *m*/*z* 191.0556, 179.0359, and 135.0448 observed in the MS spectrum were consistent with 3‐CQA [43]. In the ^1^H NMR spectrum, a pair of coupled protons at *δ* 7.58 (d, *J* = 16.0 Hz) and 6.34 (d, *J* = 16.0 Hz), along with three aromatic protons at *δ* 7.06 (d, *J* = 2.0 Hz), 6.95 (dd, *J* = 8.2, 2.0 Hz), and 6.78 (d*, J* = 8.2 Hz), confirmed the caffeoyl moiety. Additionally, the characteristic splitting pattern of the proton at *δ* 5.36 (dt, *J* = 4.2, 3.6 *H* *z*) was assigned to H‐3 of the quinic acid residue, further verifying the structure.

#### 3.1.3. Identification of 4‐CQA

The quasi‐molecular ion at *m*/*z* 353.0770 and key fragment ions at *m*/*z* 173.0483 and 179.0358 observed in the MS spectrum were consistent with 4‐CQA [44]. In the ^1^H NMR spectrum, a pair of coupled protons at *δ* 7.64 (d, *J* = 16.0 Hz) and 6.37 (d, *J* = 16.0 Hz), along with three aromatic protons at *δ* 7.07 (d, *J* = 2.0 Hz), 6.97 (dd, *J* = 8.2, 2.0 Hz), and 6.78 (d, *J* = 8.2 Hz), confirmed the caffeoyl moiety. Additionally, the proton at *δ* 4.80 (br d*, J* = 6.0 Hz) was assigned to H‐4 of the quinic acid residue, further verifying the structure as 4‐CQA.

#### 3.1.4. Identification of 3,4‐DCQA

MS analysis revealed a quasi‐molecular ion at *m*/*z* 515.1100 and a diagnostic fragment at *m*/*z* 353.0780, characteristic of a dicaffeoylquinic acid derivative [26]. Structural confirmation was provided by the ^1^H NMR data. The spectrum displayed two sets of *trans*‐olefinic proton signals at *δ* 7.58 (d, *J* = 16.0 Hz) and 6.29 (d, *J* = 16.0 Hz), and at *δ* 7.56 (d, *J* = 16.0 Hz) and 6.27 (d, *J* = 16.0 Hz). These, in conjunction with two distinct ABX‐type aromatic systems [*δ* 7.05 (d, *J* = 2.1 Hz), 6.93 (dd*, J*  = 8.1, 2.1 Hz), and 6.78 (d*, J* = 8.1 Hz); and *δ* 7.03 (d, *J* = 2.1 Hz), 6.89 (dd*, J* = 8.1, 2.1 Hz), and 6.74 (d, *J* = 8.1 Hz)], established the presence of two caffeoyl units. Furthermore, the proton signals in the mid‐range region at *δ* 5.65 (dt, *J* = 4.7, 3.1 Hz, H‐3) and *δ* 5.02 (dd, *J* = 8.6, 3.1 Hz, H‐4) exhibited splitting patterns diagnostic of the quinic acid core. Collectively, these spectral data led to the identification of the compound as 3,4‐DCQA.

#### 3.1.5. Identification of 3,5‐DCQA

MS analysis revealed a quasi‐molecular ion at *m*/*z* 515.1100 and a diagnostic fragment at *m*/*z* 353.0780, characteristic of a dicaffeoylquinic acid derivative [26]. Structural confirmation was provided by the ^1^H NMR data. The spectrum displayed two sets of *trans*‐olefinic proton signals at *δ* 7.56 (d, *J* = 16.0 Hz) and 6.25 (d, *J* = 16.0 Hz), and at *δ* 7.49 (d, *J* = 16.0 Hz) and 6.17 (d, *J* = 16.0 Hz). These, in conjunction with two distinct ABX‐type aromatic systems [*δ* 6.99 (d, *J* = 2.1 Hz), 6.89 (dd, *J* = 8.1, 2.1 Hz), and 6.72 (d, *J* = 8.1 Hz); and *δ* 6.97 (d, *J* = 2.1 Hz), 6.87 (dd, *J* = 8.1, 2.1 Hz), and 6.71 (d, *J* = 8.1 Hz)], established the presence of two caffeoyl units. Furthermore, the proton signals in the mid‐range region at *δ* 5.63 (ddd, *J* = 10.0, 9.6, 5.7 Hz, H‐5) and *δ* 4.95 (dd, *J* = 9.6, 3.1 Hz, H‐4) exhibited splitting patterns diagnostic of the quinic acid core. Collectively, these spectral data led to the identification of the compound as 3,5‐DCQA.

#### 3.1.6. Identification of 4,5‐DCQA

MS analysis revealed a quasi‐molecular ion at *m*/*z* 515.1100 and a diagnostic fragment at *m*/*z* 353.0780, characteristic of a dicaffeoylquinic acid derivative [26]. Structural confirmation was provided by the ^1^H NMR data. The spectrum displayed two sets of *trans*‐olefinic proton signals at *δ* 7.60 (d, *J* = 16.0 Hz) and 6.29 (d, *J* = 16.0 Hz), and at *δ* 7.52 (d, *J* = 16.0 Hz) and 6.20 (d, *J* = 16.0 *H* *z*). These, in conjunction with two distinct ABX‐type aromatic systems [*δ* 7.03 (d, *J* = 2.1 Hz), 6.92 (dd, *J* = 8.1, 2.1 Hz), and 6.76 (d, *J* = 8.1 Hz); and *δ* 7.01 (d, *J* = 2.1 Hz), 6.91 (dd, *J* = 8.1, 2.1 Hz), and 6.74 (d, *J* = 8.1 Hz)], established the presence of two caffeoyl units. Furthermore, the proton signals in the mid‐range region at *δ* 5.67 (ddd, *J* = 10.0, 9.6, 5.4 Hz, H‐5) and *δ* 5.12 (dd, *J* = 9.6, 3.1 Hz, H‐4) exhibited splitting patterns diagnostic of the quinic acid core. Collectively, these spectral data led to the identification of the compound as 4,5‐DCQA.

### 3.2. In Vitro Hypoglycemic Activity of CQAs

Based on the number and position of ester bonds between caffeic acid and quinic acid, CQAs are classified into mono‐, di‐, and poly‐CQAs [6]. Among these, 5‐CQA is the predominant mono‐CQA isomer [45], whereas 3,4‐DCQA, 3,5‐DCQA, and 4,5‐DCQA represent the major di‐CQA variants [6]. The multifaceted bioactivities of 5‐CQA, including antioxidant, antimicrobial, metabolic regulatory (e.g., hypoglycemic and lipid lowering), and antiviral effects, have been well‐documented [46]. In contrast, bioactivity studies on other CQA isomers remain limited. Our study evaluated the hypoglycemic effects of CQAs through *α*‐glucosidase inhibition, *α*‐amylase inhibition, glucose uptake, and glucose production assays.

#### 3.2.1. Inhibitory Effects of CQAs on *α*‐Glucosidase and *α*‐Amylase

**Figure 3:**
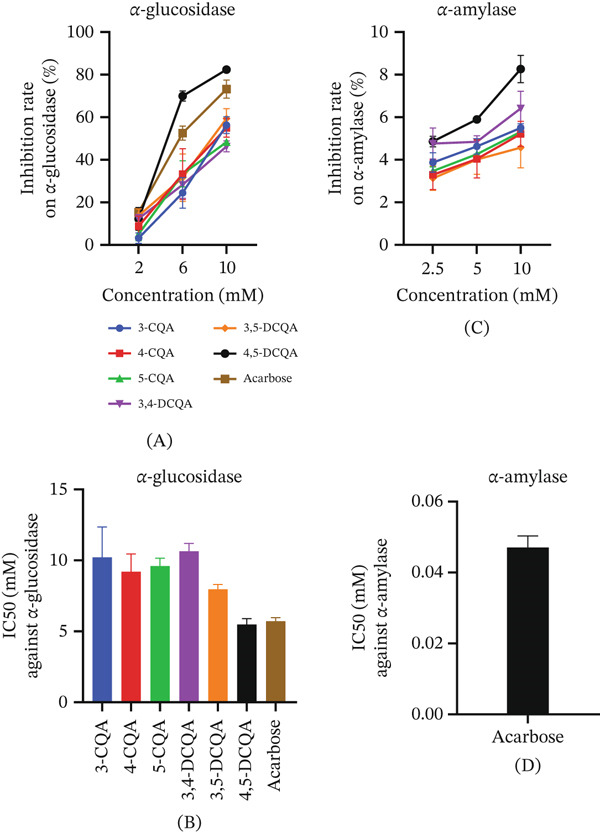
Inhibitory effects of six CQAs on *α*‐glucosidase and *α*‐amylase. (A) The inhibitory rates (%) of CQAs and the positive control acarbose against *α*‐glucosidase activity at concentrations of 2, 6, and 10 mM. (B) The IC₅₀ values of CQAs and acarbose against *α*‐glucosidase. (C) The inhibitory rates (%) of CQAs against *α*‐amylase activity at concentrations of 2.5, 5, and 10 mM. (D) The IC₅₀ value of acarbose against *α*‐amylase. Data are presented as mean ± SD (*n* = 3).

Dietary starch is hydrolyzed by *α*‐amylase into maltose and dextrin, which are subsequently converted to glucose by *α*‐glucosidase, leading to elevated blood glucose levels [47]. Inhibition of these enzymes can slow glucose release into the bloodstream, thereby helping to regulate blood sugar levels. CQAs demonstrated dose‐dependent inhibition of *α*‐glucosidase (Figure 3A), with inhibitory potency (based on IC_50_ values) following this order: 4,5‐DCQA (5.49 mM) > 3,5‐DCQA (7.96 mM) > 4‐CQA (9.21 mM) > 5‐CQA (9.60 mM) > 3‐CQA (10.22 mM) > 3,4‐DCQA (10.64 mM) (Figure 3B). Notably, 4,5‐DCQA showed comparable inhibition to that of acarbose (5.72 mM). For *α*‐amylase, all CQAs showed minimal inhibition, with less than 9% activity suppression even at 10 mM (Figure 3C). In contrast, acarbose potently inhibited *α*‐amylase with an IC₅₀ of 0.047 mM (Figure 3D).

#### 3.2.2. Effects of CQAs on HepG2 Cell Viability

**Figure 4:**
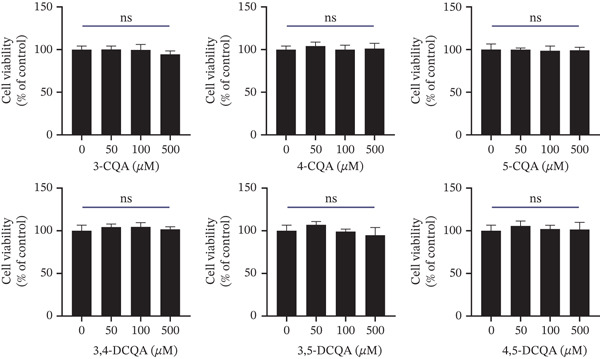
Effects of six CQAs on the viability of HepG2 cells. HepG2 cells were treated with various concentrations (50, 100, and 500 *μ*M) of each CQA for 24 h. Cell viability was assessed by the MTT assay and is expressed as a percentage of the untreated control group. Data are presented as mean ± SD (*n* = 3). ns represents no significant difference (*p* > 0.05) compared with the control group.

The cytotoxicity of CQAs was evaluated in HepG2 cells by MTT assay after 24 h of treatment. No significant cytotoxic effects were observed at any tested concentration (*p* > 0.05 vs. untreated control; Figure 4).

#### 3.2.3. Effects of CQAs on Glucose Uptake and Production

**Figure 5:**
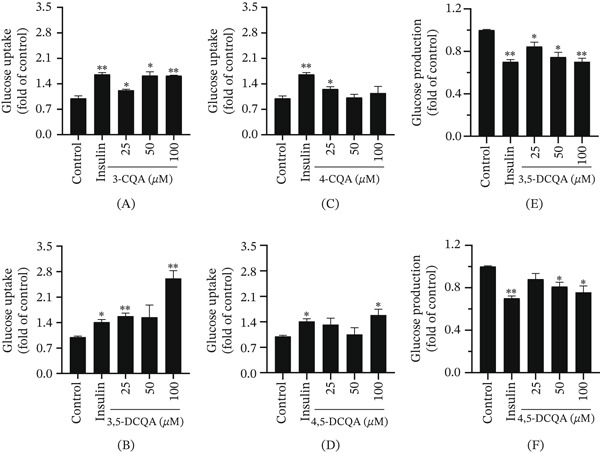
Effects of CQAs on glucose uptake and production in HepG2 cells. (A–D) Effects on glucose uptake. Cells were treated with (A) 3‐CQA, (B) 3,5‐DCQA, (C) 4‐CQA, and (D) 4,5‐DCQA at the indicated concentrations (25, 50, and 100 *μ*M). (E, F) Effects on the suppression of glucose production. Cells were treated with (E) 3,5‐DCQA and (F) 4,5‐DCQA at the indicated concentrations (25, 50, and 100 *μ*M). Insulin was used as a positive control in both assays. Data are expressed as fold change relative to the untreated control group and presented as mean ± SD (*n* = 3). ∗*p* < 0.05 and ∗∗*p* < 0.01 versus control group.

The fluorescent D‐glucose analog 2‐NBDG was used to evaluate glucose uptake [48]. Based on their *α*‐glucosidase inhibitory activities, four compounds (3‐CQA, 4‐CQA, 3,5‐DCQA, and 4,5‐DCQA) were selected for glucose uptake assays in HepG2 cells. Both 3‐CQA and 3,5‐DCQA significantly enhanced 2‐NBDG uptake in a dose‐dependent manner (25–100 *μ*M; *p* < 0.05; Figure 5A,B). Notably, 3,5‐DCQA exhibited a stronger stimulatory effect than the positive control insulin, whereas 3‐CQA (50–100 *μ*M) only reached a similar level (without surpassing insulin). Significant enhancement was also observed for 4‐CQA (25 *μ*M) and 4,5‐DCQA (100 *μ*M) (*p* < 0.05; Figure 5C,D), with the latter matching the positive control. In addition, the inhibitory effects of 3,5‐DCQA and 4,5‐DCQA on endogenous glucose production were investigated in glucose‐starved HepG2 cells. A significant suppression of endogenous glucose production by both compounds was observed (*p* < 0.05; Figure 5E,F), with the inhibitory effect of 3,5‐DCQA being significant even at a lower concentration of 25 *μ*M.

Comparative studies have established that di‐CQAs generally exhibit greater free radical scavenging capacity than mono‐CQAs, owing to their higher number of phenolic hydroxyl groups, which enhances electron‐donating capacity [6, 49]. To examine whether this structure‐activity relationship extends to antihyperglycemic effects, the *α*‐glucosidase inhibitory activity of six CQA compounds was evaluated. Consistent with the antioxidant‐based trend, the di‐CQAs 4,5‐ and 3,5‐DCQA were the most active, demonstrating greater potency than the mono‐CQAs. However, the activity of the di‐CQA isomer 3,4‐DCQA was notably lower, ranking as the least active among all six compounds. This demonstrates that antidiabetic activity is not solely determined by caffeoyl group count but is equally dependent on the specific substitution pattern. In contrast to their potent *α*‐glucosidase inhibition, CQAs exhibited negligible activity against *α*‐amylase, indicating their selectivity for inhibiting oligosaccharide breakdown rather than polysaccharide digestion. This property may regulate postprandial blood glucose levels while preserving normal starch digestion. Such selectivity may help prevent gastrointestinal adverse reactions caused by undigested starch accumulation in the colon [50]. Additionally, glucose uptake and production assays demonstrated CQAs′ dual regulatory effects on glucose metabolism. Specifically, 3,5‐DCQA dose‐dependently enhanced cellular glucose uptake while potently inhibiting hepatic glucose production even at low concentrations. These findings establish di‐CQAs, particularly 3,5‐DCQA and 4,5‐DCQA, as multitarget therapeutic candidates for T2D, with the capacity to concurrently ameliorate both fasting and postprandial hyperglycemia, the two hallmark pathological features of this disease [51].

### 3.3. Network Pharmacology Analysis Based on Component‐Target‐Pathway (CTP) Interactions

#### 3.3.1. Prediction of Potential Therapeutic Targets for CQAs

**Figure 6:**
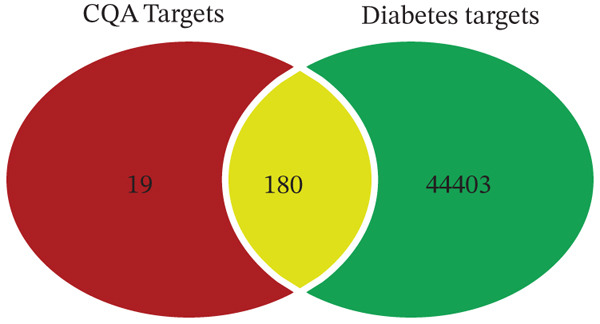
Venn diagram of the overlapping targets between CQAs and diabetes. The significant overlap (180 out of 199 CQA targets) suggests CQAs exert antidiabetic effects by modulating multiple diabetes‐associated targets.

The CAS numbers of the six CQAs were obtained from SciFinder and PubChem (Table 2). Potential targets of CQAs were predicted using both the Swiss Target Prediction and PharmMapper databases. Following removal of duplicate targets, 199 unique targets were identified. Diabetes‐related targets were identified by searching the GeneCards and OMIM databases using four relevant keywords, resulting in 44,583 diabetes‐associated targets. Comparative analysis using Venny 2.1.0 revealed 180 overlapping targets between the CQA‐related and diabetes‐related target sets, representing 90.45% of all CQA targets (Figure 6). This substantial target overlap suggests potential hypoglycemic effects of CQAs through modulation of shared biological targets.

**Table 2:** CAS numbers of six CQAs.

No.	Compound	CAS No.
C1	3‐CQA	327‐97‐9
C2	4‐CQA	905‐99‐7
C3	5‐CQA	906‐33‐2
C4	3,4‐DCQA	14534‐61‐3
C5	3,5‐DCQA	2450‐53‐5
C6	4,5‐DCQA	57378‐72‐0

#### 3.3.2. Construction and Analysis of a PPI Network

**Figure 7:**
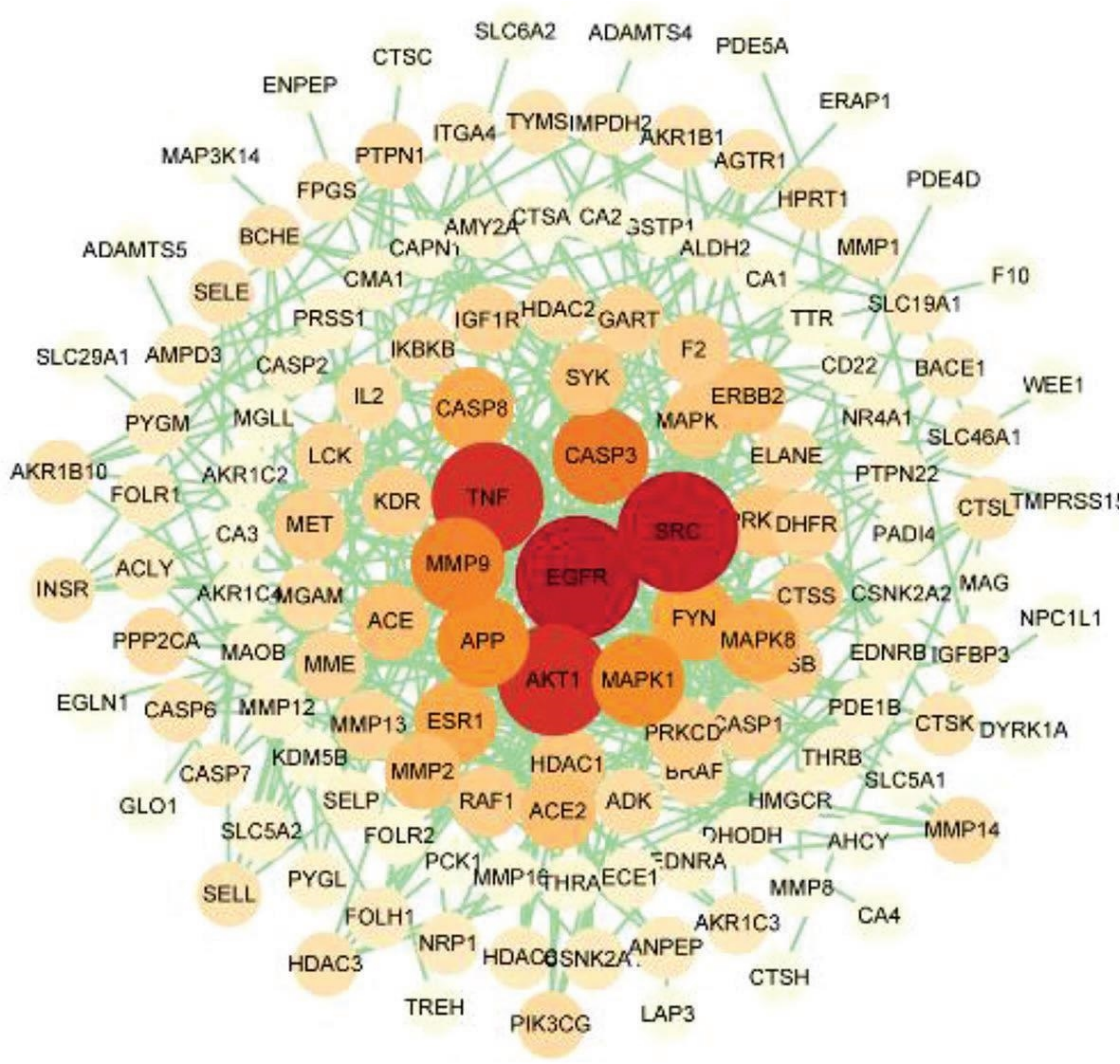
PPI network of the common targets between CQAs and diabetes. The network contains 139 nodes and 371 edges.

The PPI network of intersecting targets was constructed using the STRING database and analyzed with Cytoscape 3.9.1. The resulting network comprised 139 nodes and 371 edges (Figure 7). Topological analysis identified 10 core targets (Table 3), which encompass key molecules regulating glucose metabolic homeostasis, including core signaling molecules (e.g., AKT1), key regulators (e.g., TNF, ESR1), and regulatory components within signaling networks (e.g., SRC, FYN, EGFR, MAPK1). These collectively constitute critical targets for CQAs to regulate glucose metabolism.

**Table 3:** Top 10‐ranked hub genes in PPI network.

No.	Rank	Gene symbol	Degree value	Betweenness centrality
1	1	EGFR	31	0.20
2	2	SRC	30	0.07
3	3	AKT1	26	0.21
4	3	TNF	26	0.18
5	4	CASP3	19	0.07
6	5	MMP9	18	0.12
7	6	APP	17	0.16
8	6	MAPK1	17	0.05
9	7	FYN	14	0.04
10	8	ESR1	13	0.02

*Note:* Targets are ranked primarily by degree value, with ties resolved by betweenness centrality.

#### 3.3.3. GO and KEGG Enrichment Analysis

**Figure 8:**
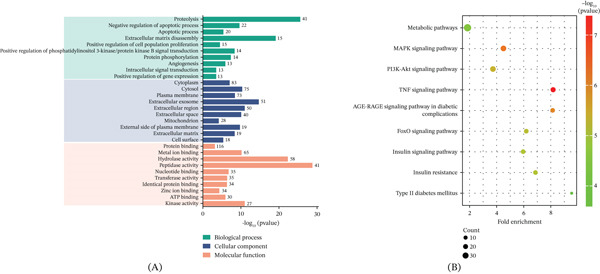
Functional enrichment analysis of the 139 CQAs targets. (A) Top 10 enriched GO terms in BP (green), CC (blue), and MF (orange). Bar length represents ‐log10(*p* value). See Tables S1–S3 for full data. (B) Key glucose metabolism‐related KEGG pathways. Bubble size indicates gene count; color indicates −log10(*p* value). See Tables S4 for full data.

GO enrichment analysis was performed on the 139‐screened potential targets using DAVID 2021, with thresholds set at count ≥ 10 and FDR ≤ 0.01. The Top 10 significantly enriched terms in the three GO categories of biological processes (BP), molecular functions (MF), and cellular components (CC) are visualized in Figure 8A, with complete data available in Tables S1–S3. The top‐ranked BP terms were closely associated with diabetes pathophysiology, including extracellular matrix disassembly, positive regulation of cell population proliferation, negative regulation of apoptotic process, and notably, positive regulation of the phosphatidylinositol 3‐kinase/protein kinase B (PI3K/AKT) signaling pathway. At the molecular level, significant MF enrichment was found in kinase activity, ATP binding, metal ion binding, and peptidase activity, indicating a capacity for signal transduction and protein modification. CC analysis showed predominant localization to the plasma membrane, cytoplasm, and extracellular region, consistent with roles in signal reception and intercellular communication.

Parallel KEGG pathway analysis (count ≥ 5, FDR ≤ 0.01) identified 99 significantly enriched pathways (p < 0.01), with nine specifically associated with glucose metabolism: metabolic pathways, MAPK signaling pathway, PI3K/AKT signaling pathway, TNF signaling pathway, AGE‐RAGE signaling pathway in diabetic complications, FoxO signaling pathway, insulin signaling pathway, insulin resistance, and T2D mellitus (Figure 8B, Table S4).

Given the well‐established role of the PI3K/AKT pathway in insulin signaling and glucose homeostasis [52], its significant enrichment in our analyses suggests that modulating this pathway is a central mechanism by which the core targets of CQAs improve insulin sensitivity and hepatic glucose metabolism.

#### 3.3.4. Construction and Analysis of a CTP Network

**Figure 9:**
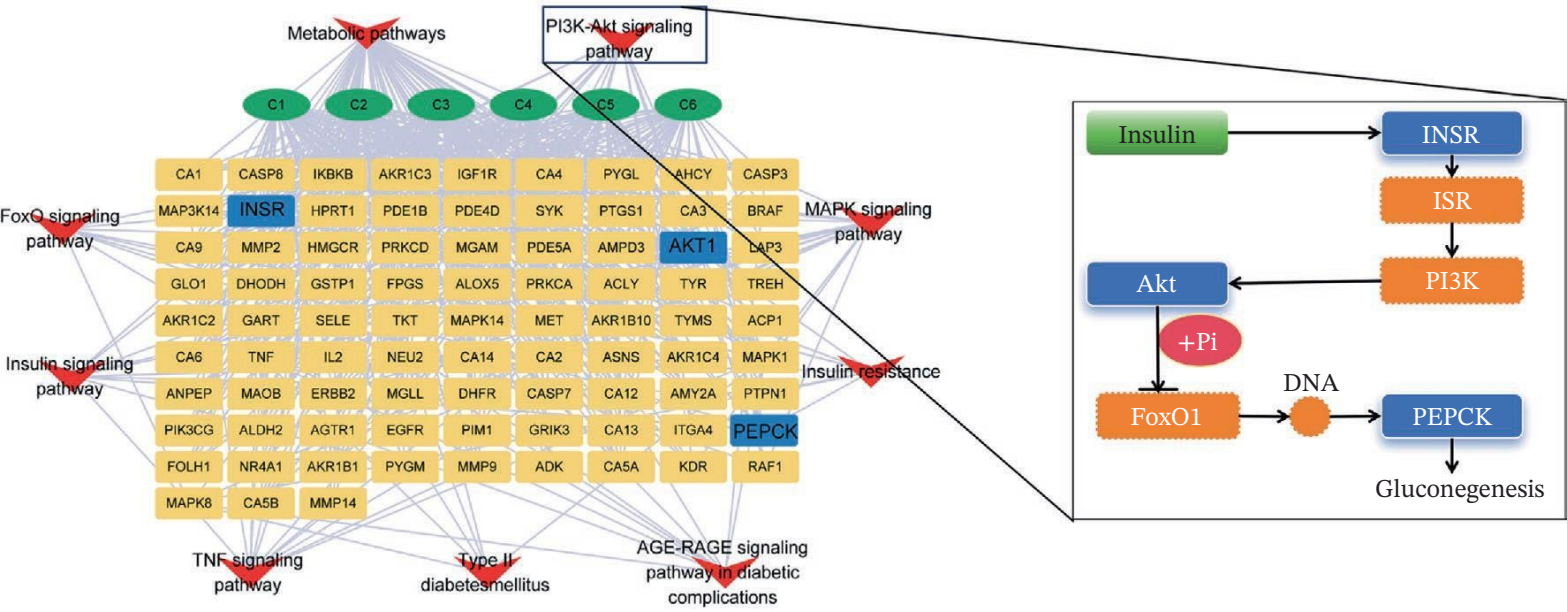
CTP network illustrates the multitarget mechanism of CQAs regulating glucose homeostasis. The integrated network (99 nodes, 434 edges) connects six CQA compounds with enriched KEGG pathways and their targets. Analysis identifies AKT1, PEPCK, and INSR as central hubs, highlighting the PI3K/AKT signaling pathway′s role in suppressing gluconeogenesis via PEPCK.

The CTP network (Figure 9) was constructed in Cytoscape 3.9.1 by integrating all six CQA compounds with nine blood glucose‐regulating pathways and their corresponding targets from KEGG enrichment analysis, resulting in a network of 99 nodes and 434 edges. Three protein targets, AKT1, PEPCK, and INSR, emerged as central hubs, and the PI3K/AKT, insulin, and FoxO signaling pathways showed stronger associations with CQA activity, suggesting their potential importance in mediating the observed effects.

In the pathogenesis of T2D, dysregulation of hepatic glucose metabolism arises from the dual defects of impaired insulin signaling and excessive gluconeogenic activation. A major contributor to this process is the pathological overexpression of PEPCK, the rate‐limiting enzyme in gluconeogenesis [53]. Under physiological conditions, insulin binding to its receptor activates the IRS‐1/PI3K/AKT signaling cascade, leading to AKT‐mediated phosphorylation and nuclear exclusion of FOXO1, while simultaneously suppressing CREB transcriptional activity, both of which are critical mechanisms for maintaining gluconeogenic control [54, 55]. In the diabetic state, however, proinflammatory signaling induces serine phosphorylation of IRS‐1, impairing PI3K/AKT activation. This results in nuclear accumulation of active FOXO1 and loss of CREB suppression, which synergistically drive PEPCK gene transcription [53]. Therapeutic strategies targeting either PEPCK inhibition or restoration of the insulin signaling pathway components may therefore offer novel approaches for diabetes treatment.

### 3.4. CQAs Inhibit Gluconeogenesis via Targeting PEPCK and Insulin Signaling

**Figure 10:**
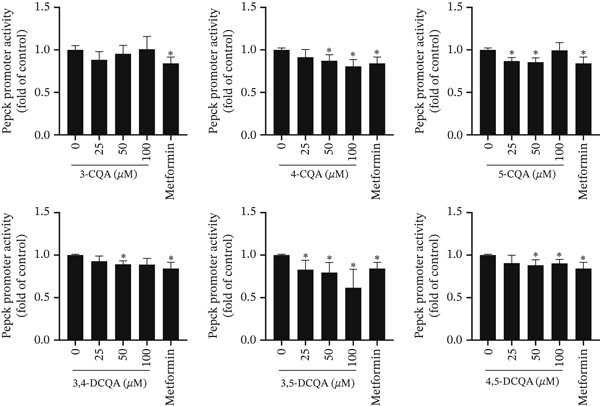
Inhibitory effects of CQAs on *Pepck* promoter activity. HepG2 cells were treated with increasing concentrations (0–100 *μ*M) of six CQAs or metformin (positive control) for 24 h. Promoter activity was measured by dual‐luciferase reporter assay and is presented as fold change relative to the untreated control group. Data are presented as mean ± SD (*n* = 3). ∗*p* < 0.05 versus control group.

A dual‐luciferase reporter assay revealed that all CQA compounds except 3‐CQA significantly suppressed *Pepck* promoter activity (*p* < 0.05). Among these, 4‐CQA and 3,5‐DCQA demonstrated concentration‐dependent inhibition within the 25–100 *μ*M range. At the highest tested concentration (100 *μ*M), both compounds exhibited stronger inhibitory effects than metformin, the positive control (Figure 10). These results indicate that specific CQA derivatives may regulate hepatic glucose production through PEPCK‐mediated suppression of gluconeogenesis.

**Figure 11:**
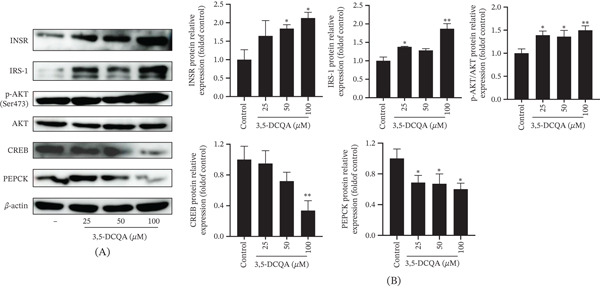
Mechanisms of 3,5‐DCQA in regulating hepatic glucose metabolism. (A) Representative western blot images of INSR, IRS‐1, p‐AKT, AKT, CREB, and PEPCK in HepG2 cells treated with 3,5‐DCQA (0–100 *μ*M). (B) Quantification of (A) from three independent experiments. *β*‐Actin was used as a loading control. Data are presented as fold change relative to control (mean ± SD; ∗*p* < 0.05, *p* < 0.01 versus control).

Building on previous findings, 3,5‐DCQA was selected for western blot analysis of key gluconeogenic pathway proteins (Figure 11). 3,5‐DCQA treatment concentration‐dependently upregulated insulin signaling components, significantly enhancing INSR, IRS‐1, and p‐AKT/AKT expression (*p* < 0.05). Concurrently, it suppressed CREB (significant at 100 *μ*M, *p* < 0.01) and PEPCK expression (at all concentrations, *p* < 0.05).

Our results demonstrated that 3,5‐DCQA suppressed PEPCK through both transcriptional and posttranscriptional mechanisms, specifically by reducing its promoter activity and decreasing protein abundance. The observed decrease in CREB protein further supports the compound′s role in suppressing gluconeogenic signaling. Beyond this direct suppression of gluconeogenesis, 3,5‐DCQA also enhanced insulin sensitivity, which was evidenced by its upregulation of key insulin signaling molecules. Specifically, treatment with 3,5‐DCQA significantly elevated INSR and IRS‐1 protein expression and enhanced AKT phosphorylation (p‐AKT), suggesting its multitarget regulatory role in glucose homeostasis. The absence of p‐IRS‐1 measurements means we cannot definitively conclude that 3,5‐DCQA engages the canonical insulin signaling pathway. Therefore, the observed activation of p‐AKT could result from alternative mechanisms, such as direct modulation of AKT or other IRS‐1‐independent pathways, which contribute to its glucose‐lowering effects. Notably, the observed activation of the INSR/AKT axis and suppression of PEPCK directly validate the key targets predicted by network pharmacology. The additional finding of reduced CREB protein, which was not among the key targets predicted by network pharmacology, offers further mechanistic insight into the transcriptional inhibition of gluconeogenesis.

Taken together, our results delineate a multitarget mechanism of action for 3,5‐DCQA, which involves the concurrent suppression of the CREB/PEPCK axis and potentiation of the INSR/IRS‐1/AKT signaling pathway. This mechanism differs from that reported for 5‐CQA, which has been shown to primarily involve the AMPK pathway and subsequent suppression of G6Pase [56–58]. Collectively, these findings demonstrate that different CQA derivatives can utilize distinct pathways to combat hyperglycemia.

## 4. Conclusion

*L. japonica* Thunb., a dual‐purpose medicinal and edible resource, is a rich source of CQAs. This study identified six CQAs from *L. japonica*, among which 3,5‐DCQA demonstrated the most promising profile in our pharmacological evaluation. Integrated network pharmacology and experimental analyses revealed that 3,5‐DCQA exerts multitarget effects against hyperglycemia, primarily by suppressing hepatic gluconeogenesis and potentiating insulin signaling, thereby highlighting the research value of modulating multiple nodes in glucose homeostasis. Collectively, our in vitro results provide compelling evidence supporting 3,5‐DCQA as a candidate worthy of further investigation as a potential lead compound. However, its therapeutic potential, precise in vivo efficacy, and long‐term safety require definitive validation through comprehensive animal studies.

## Author Contributions

**Yiyi Ye**: methodology, formal analysis, investigation, data curation, writing—original draft, writing—review and editing, and visualization. **Dawei Xu**: methodology, validation, data Curation, writing—review and editing, and visualization. **Shilu Zhang:** methodology, data curation, writing—review and editing, and visualization. **Huawei Yu**: methodology, formal analysis, investigation, and writing—original draft. **Yingwei Liu**: validation, data curation, and visualization. **Jin Yang**: conceptualization and funding acquisition. **Lijuan Sun**: conceptualization, formal analysis, resources, writing—review and editing, supervision, project administration, and funding acquisition. Yiyi Ye and Dawei Xu contributed equally as first authors.

## Funding

This study was supported by The National Natural Science Foundation of China (Nos. 31400304, 81400791, and 82174449) and Competitive research fund of Hubei University for Nationalities (XN2314).

## Conflicts of Interest

The authors declare no conflicts of interest.

## Supporting Information

Additional supporting information can be found online in the Supporting Information section.

## Supporting information


**Supporting Information 1** File S1 (Spectra): ^1^H NMR spectra for the six isolated CQAs (Figures S1–S6).

**Supporting Information 2** File S2 (Tables): Supporting tables for the GO and KEGG pathway enrichment analyses (Tables S1–S4).

## Data Availability

The data that support the findings of this study are available from the corresponding author upon reasonable request.
