# Supplementary material for: Caffeoylquinic Acids From Lonicera japonica Thunb. as Hypoglycemic Agents: Network Pharmacology and Pharmacological Validation
Source: J Diabetes Res. 2026 May 30;2026:6712215. doi: 10.1155/jdr/6712215 (PMC13239056; doi:10.1155/jdr/6712215)

Supplementary Figure S1. The ¹H NMR spectrum of 3-O-caffeoylquinic acid (3-CQA).

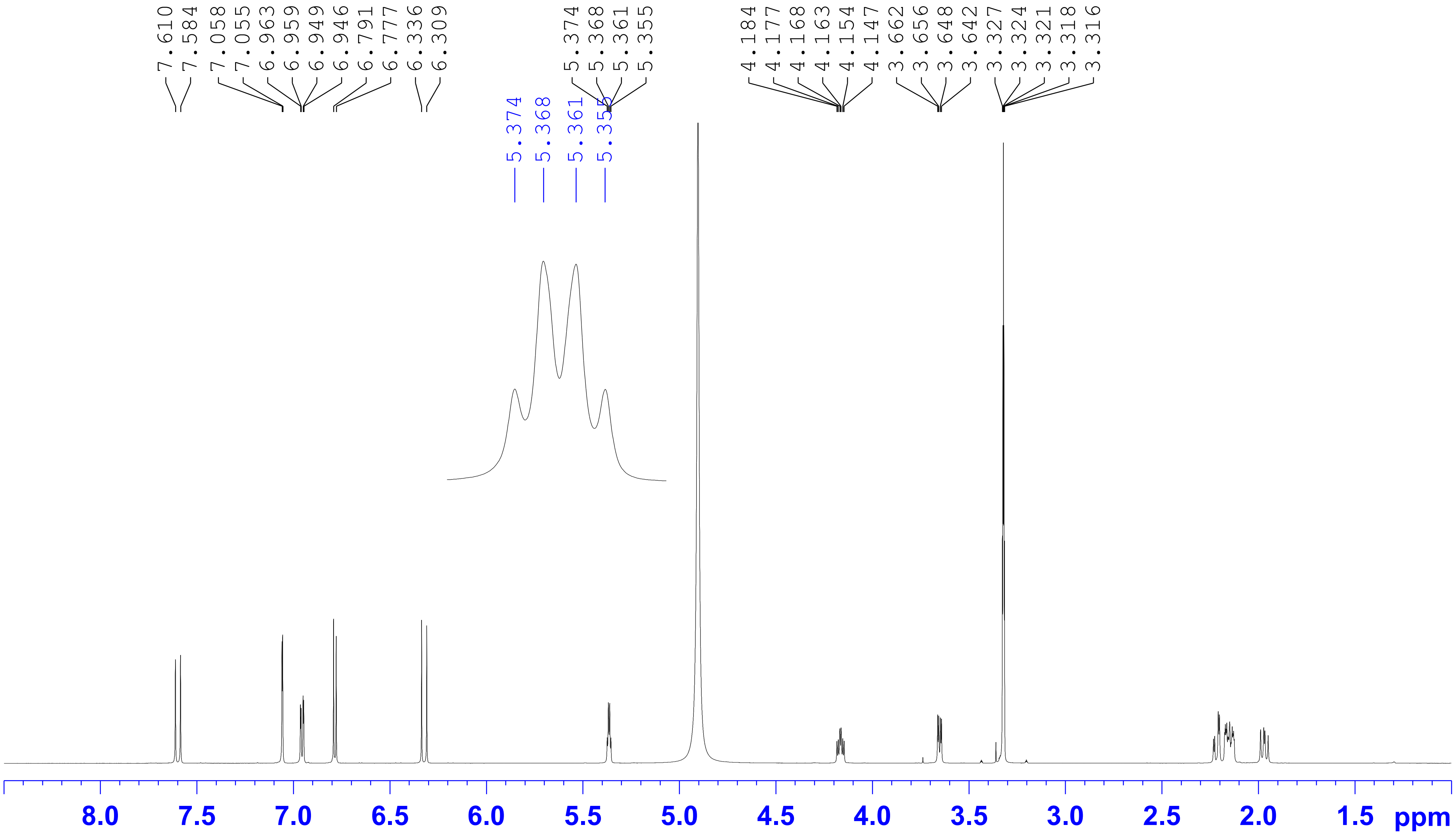

Supplementary Figure S2. The ^{1}H NMR spectrum of 5-O-caffeoylquinic acid (5-CQA).

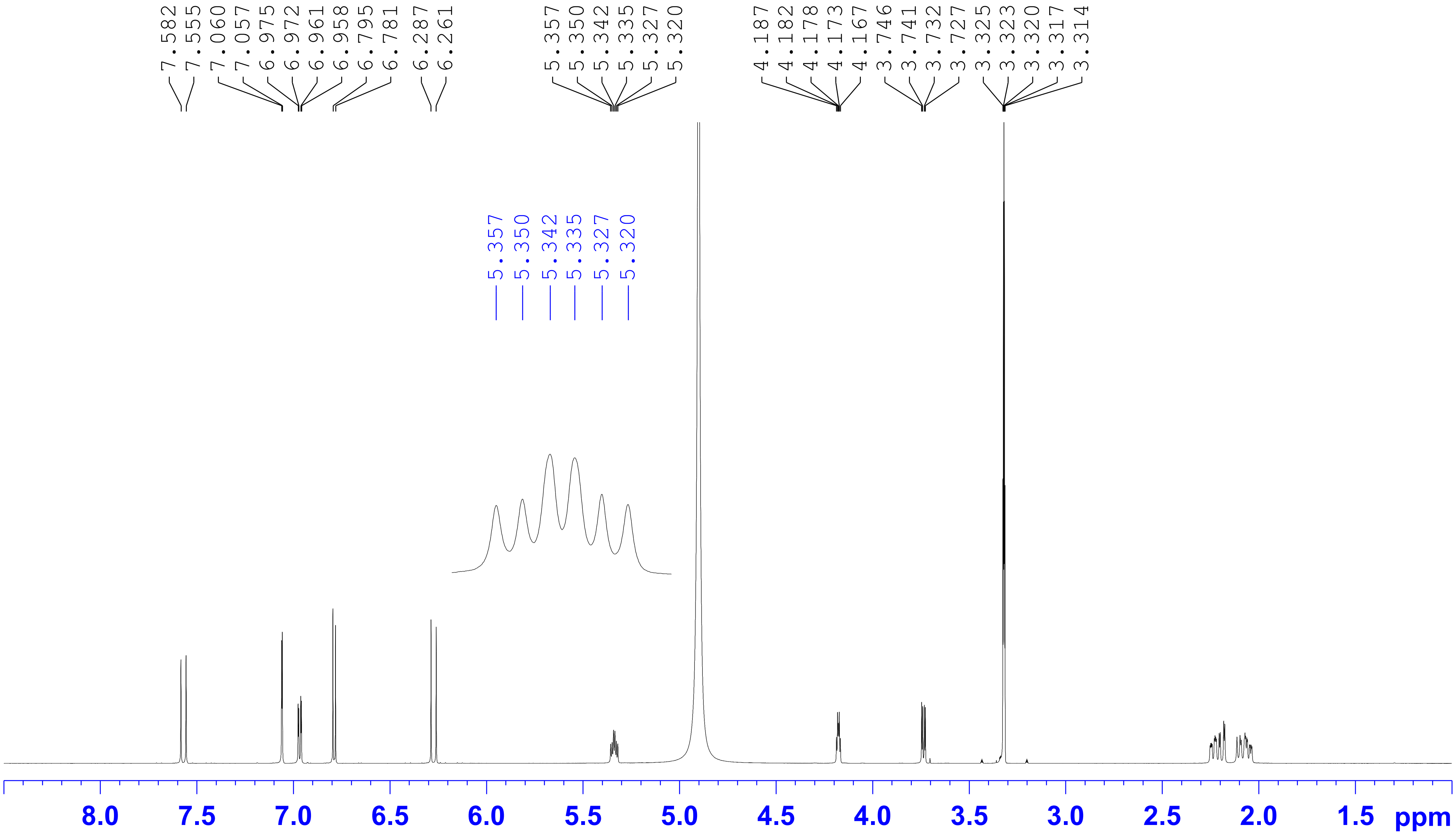

Supplementary Figure S3. The ¹H NMR spectrum of 4-O-caffeoylquinic acid (4-CQA).

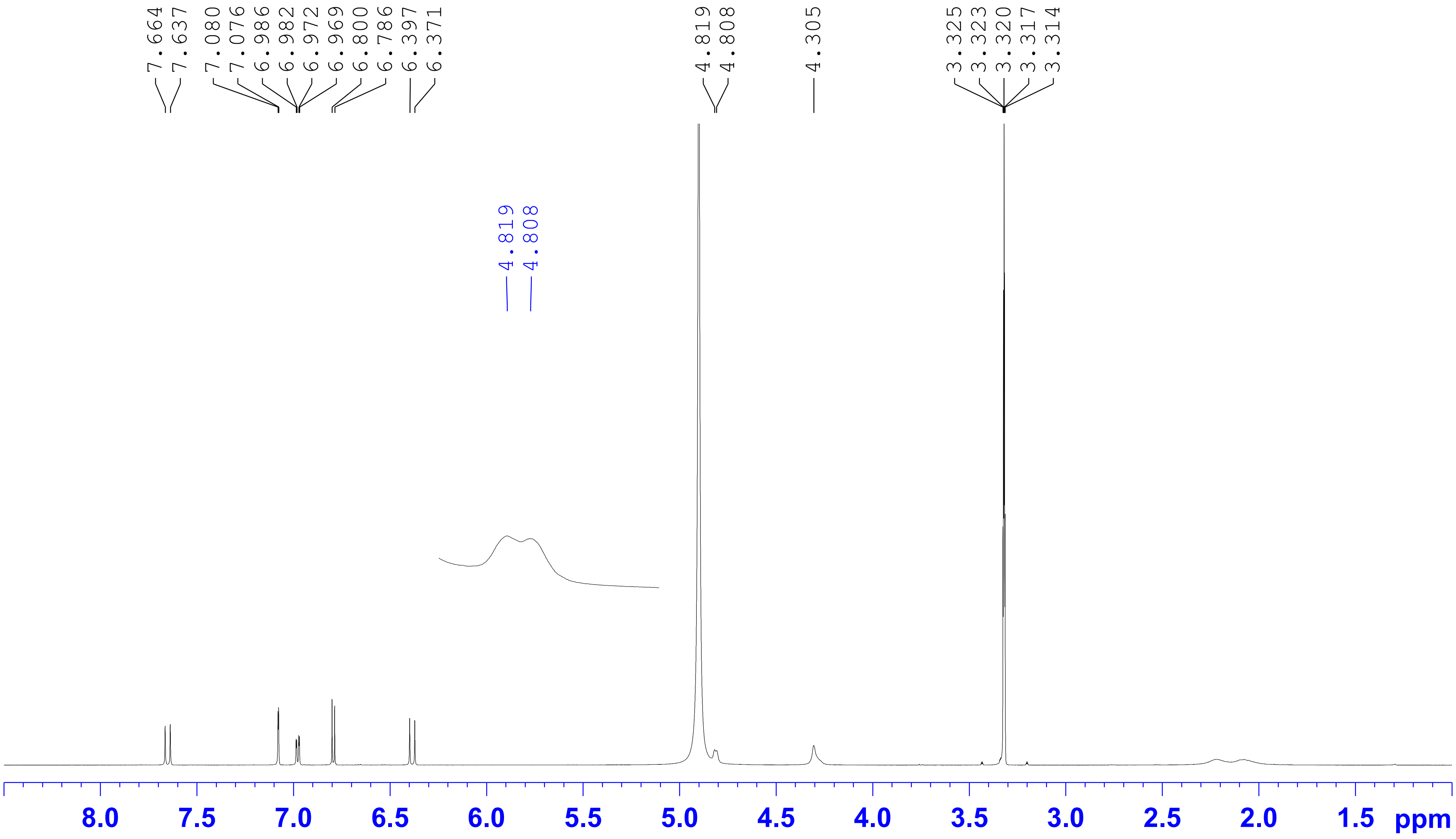

Supplementary Figure S4. The ¹H NMR spectrum of 3,4-di-O-caffeoylquinic acid (3,4-DCQA).

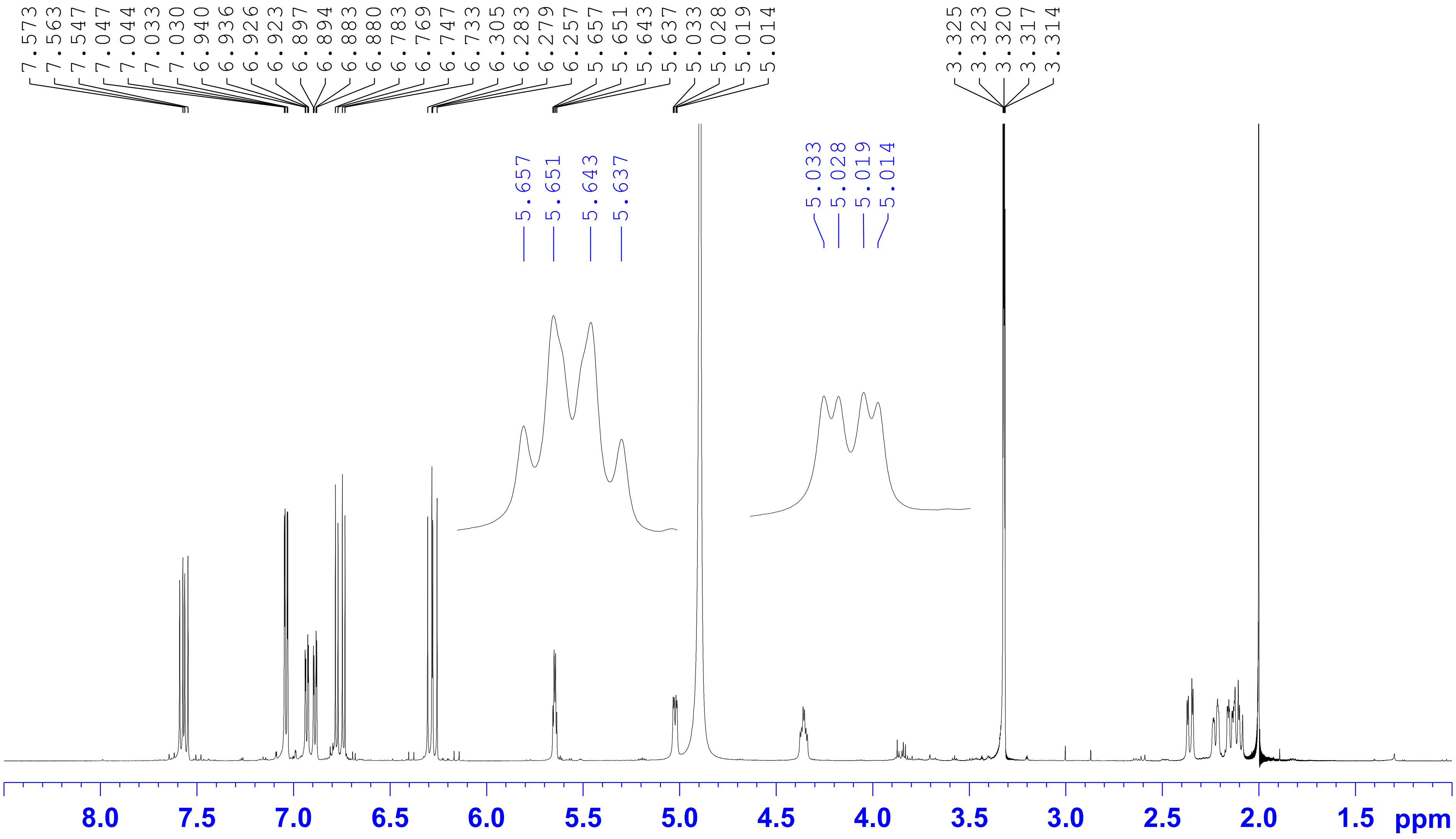

Supplementary Figure S5. The ^{1}H NMR spectrum of 4,5-di-O-caffeoylquinic acid (4,5-DCQA).

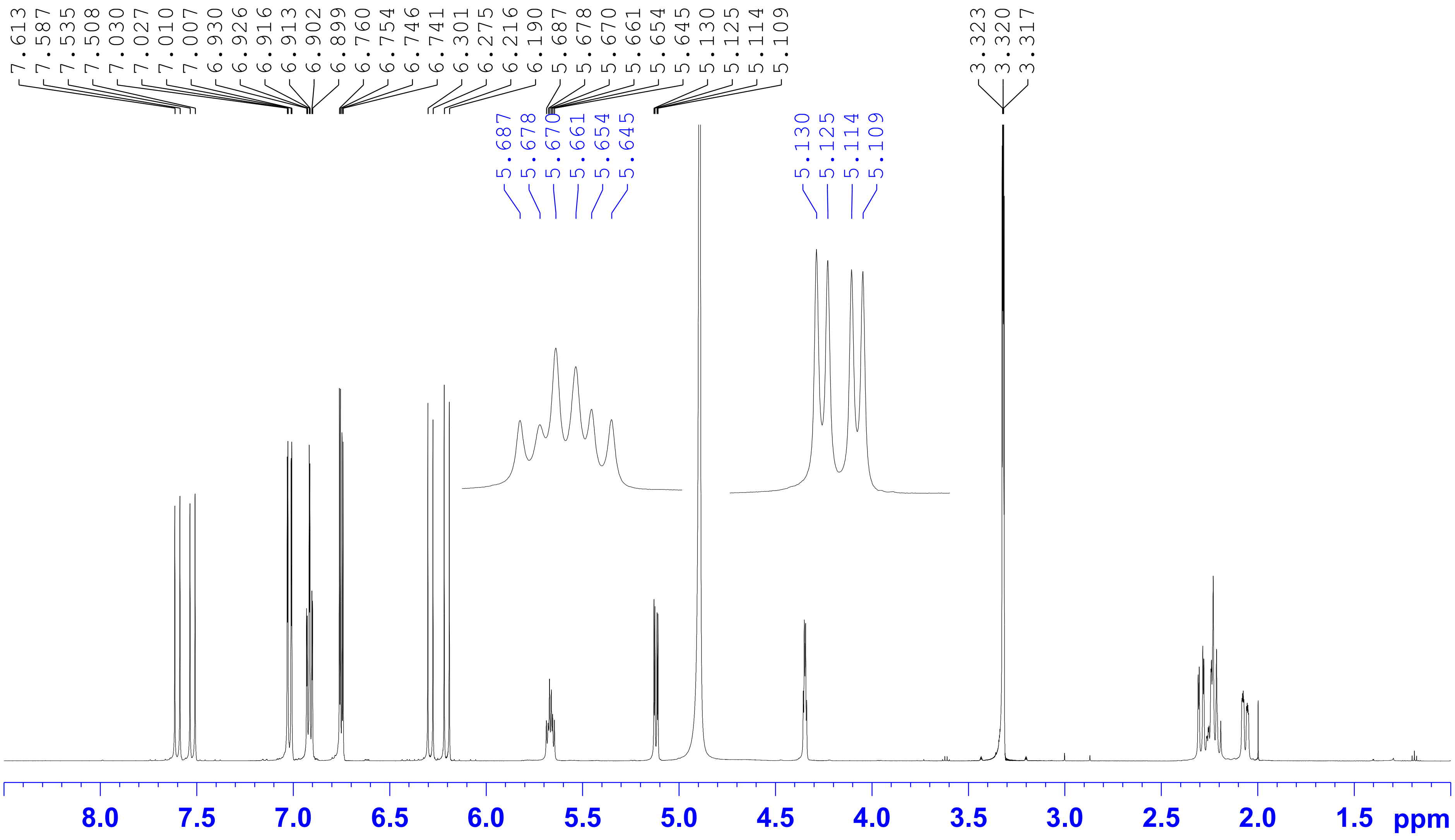

Supplementary Figure S6. The ¹H NMR spectrum of 3,5-di-O-caffeoylquinic acid (3,5-DCQA).

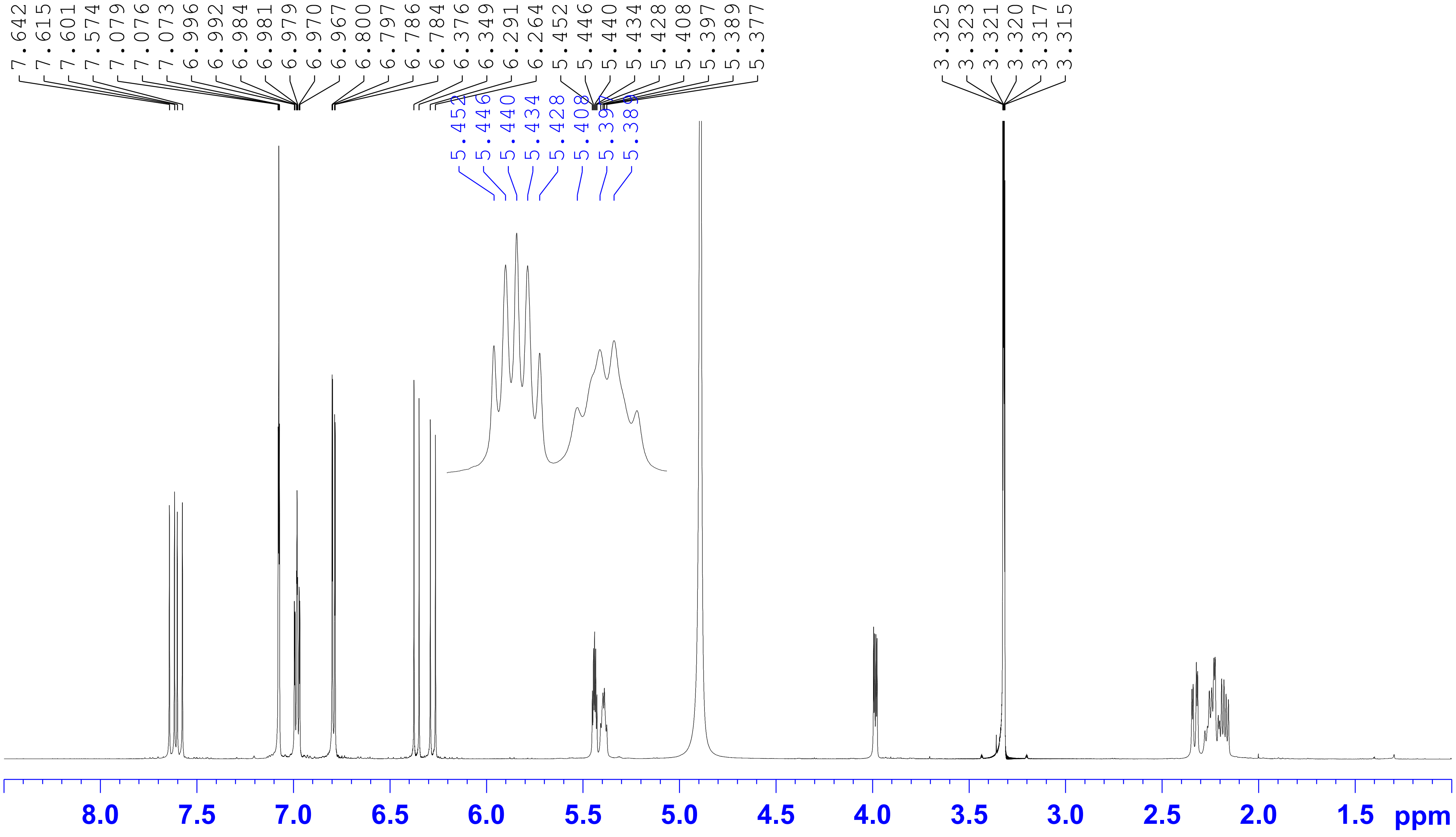

Supplement: Supplementary file 1 — Supporting Information 1 File S1 (Spectra): 1H NMR spectra for the six isolated CQAs (Figures S1–S6). [file JDR-2026-6712215-s001.pdf]
