# Supplementary material for: Caffeoylquinic Acids From Lonicera japonica Thunb. as Hypoglycemic Agents: Network Pharmacology and Pharmacological Validation
Source: J Diabetes Res. 2026 May 30;2026:6712215. doi: 10.1155/jdr/6712215 (PMC13239056; doi:10.1155/jdr/6712215)
Supplement: Supplementary file 2 — Supporting Information 2 File S2 (Tables): Supporting tables for the GO and KEGG pathway enrichment analyses (Tables S1–S4). [file JDR-2026-6712215-s002.pdf]

Supplementary Table S1. Top 10 enriched Gene Ontology (GO) biological processes.

GO ID	GO Term (BP)	Gene Count	p-value	Adjusted p-value (FDR)	Fold Enrichment	Genes
GO:0006508	proteolysis	41	3.22E-26	5.98E-23	8.6	APP,PRSS1,ENPEP,ECE1,CTSS,ADAMTS4,ADAMTS5,CASP7,CASP8,CASP6,CTSL,CASP3,ANPEP,CTSK,CASP1,CASP2,CTSH,CAPN1,ELANE,CTSC,CTSB,CTSA,ACE,MME,F10,MMP1,CMA1,MMP2,ERAP1,MMP8,F2,MMP9,TMPRSS15,BACE1,MMP12,ACE2,MMP14,MMP13,FOLH1,MMP16,LAP3
GO:0043066	negative regulation of apoptotic process	22	2.44E-10	9.06E-08	5.7	HDAC2,HDAC3,HDAC1,SRC,GSTP1,GLO1,PRKCD,AKR1B1,BRAF,TNF,MMP9,EGFR,IL2,IGF1R,MAPK8,EDNRB,CA3,ERBB2,KDR,CASP2,AKT1,RAF1
GO:0006915	apoptotic process	20	3.67E-06	2.53E-04	3.5	APP,CSNK2A1,IGFBP3,PRKCD,CSNK2A2,PRKCA,MAPK14,MMP9,NR4A1,CASP7,CASP8,CASP6,CASP3,CASP1,CASP2,CTSH,AKT1,MAPK1,RAF1,CTSC
GO:0022617	extracellular matrix disassembly	15	7.78E-20	7.23E-17	45.8	PRSS1,MMP1,CMA1,MMP2,MMP8,MMP9,CTSS,MMP12,ADAMTS4,ADAMTS5,MMP14,MMP13,CTSL,CTSK,ELANE
GO:0008284	positive regulation of cell population proliferation	15	3.09E-05	1.40E-03	3.9	HDAC2,CSNK2A1,HDAC1,INSR,AKR1C3,AKR1C2,F2,EGFR,IL2,IGF1R,EDNRB,CA3,KDR,CAPN1,FOLR2
GO:0051897	positive regulation of phosphatidylinositol 3-kinase/protein kinase B signal transduction	14	4.83E-09	1.12E-06	9.1	APP,SRC,INSR,AKR1C3,AKR1C2,PRKCA,F2,TNF,EGFR,PIK3CG,IGF1R,SELP,KDR,FYN
GO:0006468	protein phosphorylation	14	4.35E-08	6.23E-06	7.5	SYK,IGFBP3,PRKCD,DYRK1A,BRAF,PRKCA,IKBKB,MAPK8,LCK,ERBB2,AKT1,MAPK1,FYN,RAF1
GO:0001525	angiogenesis	13	1.15E-06	9.68E-05	6.3	NRP1,ENPEP,SYK,MMP2,ERAP1,PRKCA,MAPK14,PIK3CG,MMP14,EDNRA,CASP8,ANPEP,KDR
GO:0035556	intracellular signal transduction	13	2.35E-04	6.23E-03	3.6	SYK,SRC,PRKCD,PRKCA,MAPK14,PPP2CA,LCK,CASP3,ERBB2,AKT1,MAPK1,FYN,MAP3K14
GO:0010628	positive regulation of gene expression	13	3.01E-04	7.37E-03	3.5	KDM5B,APP,HDAC1,BRAF,PTPN22,MAPK14,TNF,MMP12,MAPK8,LCK,ERBB2,CTSH,AKT1

Supplementary Table S2. Top 10 enriched Gene Ontology (GO) cellular components.

GO ID	GO Term (CC)	Gene Count	p-value	Adjusted p-value (FDR)	Fold Enrichment	Genes
GO:0005737	cytoplasm	83	9.53E-08	2.07E-06	1.60	APP,NRP1,SLC46A1,THRA,ADK,AKR1B1,PYGM,PTPN22,PYGL,PIK3CG,IKBKB,CASP7,CA1,CASP8,CA3,CASP6,CA2,ANPEP,CASP3,FPGS,CASP1,KDR,CASP2,AKT1,CAPN1,CTSC,SYK,MME,PDE4D,MMP2,CSNK2A2,PRKCD,DYRK1A,PRKCA,MMP12,ACE2,ACLY,MMP14,FOLH1,LCK,PADI4,HPRT1,LAP3,RAF1,MGLL,ENPEP,HDAC2,HDAC3,AHCY,PDE1B,SRC,HDAC1,GSTP1,GLO1,TYMS,HDAC6,EGFR,PPP2CA,MAPK8,TTR,ERBB2,MAPK1,FYN,PCK1,ELANE,EGLN1,PTPN1,ACE,ERAP1,AKR1C3,AKR1C2,BRAF,AKR1C4,MAPK14,ESR1,DHODH,DHFR,NR4A1,WEE1,AKR1B10,IMPDH2,MAP3K14,CD22
GO:0005829	cytosol	75	5.16E-11	3.89E-09	1.98	KDM5B,APP,NRP1,THRA,ADK,AKR1B1,PYGM,PTPN22,PYGL,PIK3CG,IKBKB,CASP7,CA1,CASP8,CA3,CASP6,CA2,CASP3,FPGS,CASP1,CASP2,AKT1,CTSH,CAPN1,CSNK2A1,SYK,MME,PDE4D,CSNK2A2,PRKCD,DYRK1A,PRKCA,AMPD3,ACLY,MMP14,LCK,PDE5A,PADI4,HPRT1,RAF1,GART,MGLL,HDAC3,AHCY,PDE1B,SRC,HDAC1,GSTP1,GLO1,TYMS,HDAC6,EGFR,PPP2CA,MAPK8,ERBB2,MAPK1,FYN,PCK1,ELANE,EGLN1,PTPN1,CMA1,ERAP1,AKR1C3,AKR1C2,BRAF,AKR1C4,MAPK14,ESR1,DHODH,DHFR,NR4A1,AKR1B10,IMPDH2,MAP3K14
GO:0005886	plasma membrane	73	3.75E-09	1.06E-07	1.84	APP,NRP1,SLC46A1,TREH,ADK,ECE1,SLC6A2,TNF,PIK3CG,IGF1R,CASP7,EDNRA,CASP8,EDNRB,CTSL,CA2,ANPEP,CTSK,CA4,CASP1,KDR,AKT1,CAPN1,CTSB,CSNK2A1,SYK,MME,ITGA4,PDE4D,MMP2,PRKCD,PRKCA,F2,BACE1,ACE2,MAG,MMP14,FOLH1,MMP16,LCK,AGTR1,RAF1,SLC29A1,MET,MGLL,ENPEP,HDAC3,SRC,GLO1,SLC5A1,SLC5A2,EGFR,PPP2CA,MAPK8,NPC1L1,ERBB2,MAPK1,FYN,SLC19A1,PTPN1,BCHE,MGAM,ACE,F10,INSR,BRAF,SELE,ESR1,SELP,SELL,FOLR2,FOLR1,CD22
GO:0070062	extracellular exosome	51	2.11E-15	4.76E-13	3.41	APP,AMY2A,TREH,AKR1B1,PYGM,ECE1,PYGL,CA1,ALDH2,CTSL,CA2,ANPEP,CA4,CTSH,CAPN1,CTSC,CTSB,MME,ITGA4,PRKCD,PRKCA,F2,MMP9,ACE2,ACLY,FOLH1,LCK,HPRT1,LAP3,GART,ENPEP,AHCY,SRC,GSTP1,GLO1,SLC5A1,SLC5A2,PPP2CA,TTR,PCK1,ELANE,CTSA,MGAM,ACE,INSR,ERAP1,AKR1C3,AKR1C4,IMPDH2,FOLR1,CD22
GO:0005576	extracellular region	50	1.03E-11	1.16E-09	2.78	APP,NRP1,PRSS1,AMY2A,PYGL,TNF,CTSS,ADAMTS4,ADAMTS5,CASP7,CTSL,CTSK,KDR,CTSH,CAPN1,CTSC,CTSB,MME,MMP1,IGFBP3,MMP2,PRKCD,AMPD3,MMP8,F2,MMP9,MMP12,ACE2,ACLY,MMP13,MMP16,MET,GSTP1,EGFR,TTR,MAPK1,ELANE,CTSA,BCHE,MGAM,ACE,F10,CMA1,ERAP1,MAPK14,IL2,AKR1B10,IMPDH2,FOLR2,FOLR1
GO:0005615	extracellular space	40	8.05E-11	4.55E-09	3.16	APP,NRP1,PRSS1,ENPEP,AMY2A,GSTP1,AKR1B1,TNF,EGFR,CTSS,ADAMTS4,ADAMTS5,CASP7,TTR,CTSL,ANPEP,CTSK,CTSH,ELANE,CTSC,CTSB,BCHE,ACE,F10,CMA1,MMP2,IGFBP3,ERAP1,MMP8,F2,SELE,MMP9,IL2,SELP,MMP12,ACE2,MMP14,MMP13,SELL,MMP16
GO:0005739	mitochondrion	28	6.25E-05	7.84E-04	2.29	APP,NRP1,MAOB,SRC,GSTP1,AKR1B1,TYMS,PPP2CA,MAPK8,CASP8,ALDH2,FPGS,AKT1,MAPK1,FYN,CAPN1,PCK1,MMP2,PRKCD,PRKCA,BRAF,MAPK14,DHODH,DHFR,NR4A1,AKR1B10,LAP3,RAF1
GO:0009897	external side of plasma membrane	19	1.87E-10	8.46E-09	7.08	ENPEP,ACE,F10,ITGA4,INSR,ECE1,F2,SELE,TNF,SELP,SELL,ANPEP,CTSK,CA4,KDR,FOLR2,FOLR1,CD22,CTSB

GO:0031012	extracellular matrix	19	4.63E-09	1.16E-07	5.78	PRSS1,MMP1,CMA1,MMP2,F2,MMP8,MMP9,CTSS,MMP12,ADAMTS4,ADAMTS5,MMP14,MMP13,MMP16,CTSL,CTSH,ELANE,CTSC,CTSB
GO:0009986	cell surface	18	4.11E-06	6.64E-05	3.84	APP,SLC46A1,MME,ITGA4,SLC6A2,TNF,EGFR,BACE1,ACE2,FOLH1,MMP16,CA4,KDR,FOLR2,MET,FOLR1,CD22,ELANE

Supplementary Table S3. Top 10 enriched Gene Ontology (GO) molecular functions.

GO ID	GO Term (MF)	Gene Count	p-value	Adjusted p-value (FDR)	Fold Enrichment	Genes
GO:0005515	protein binding	116	7.23E-04	5.95E-03	1.17	APP,TNF,IGF1R,EDNRA,EDNRB,KDR,AKT1,CAPN1,CSNK2A1,IGFBP3,CSNK2A2,PRKCD,DYRK1A,PRKCA,ACLY,ACE2,FOLH1,AGTR1,PADI4,LAP3,HPRT1,RAF1,MAOB,PDE1B,GLO1,SLC5A1,SLC5A2,NPC1L1,SLC19A1,ELANE,MGAM,ACE,INSR,BRAF,SELE,ESR1,IL2,TMPRSS15,SELP,DHFR,NR4A1,AKR1B10,SELL,MAP3K14,CD22,KDM5B,NRP1,THRB,THRA,AKR1B1,PTPN22,ECE1,PYGM,PYGL,SLC6A2,PIK3CG,CTSS,ADAMTS4,IKBKB,ADAMTS5,CASP7,CA1,CASP8,CA3,CASP6,CTSL,CA2,CASP3,CTSK,CA4,CASP1,CASP2,CTSH,CTSC,CTSB,ITGA4,SYK,MME,PDE4D,MMP2,AMPD3,F2,MMP9,BACE1,MMP14,LCK,PDE5A,MET,MGLL,HDAC2,HDAC3,AHCY,SRC,HDAC1,GSTP1,HMGCR,HDAC6,EGFR,PPP2CA,MAPK8,TTR,ERBB2,MAPK1,FYN,EGLN1,BCHE,PTPN1,F10,ERAP1,AKR1C3,AKR1C4,MAPK14,DHODH,WEE1,IMPDH2,FOLR1
GO:0046872	metal ion binding	65	6.20E-11	2.18E-09	2.18	KDM5B,APP,NRP1,PRSS1,THRB,AMY2A,THRA,ADK,ECE1,SLC6A2,ADAMTS4,ADAMTS5,CA1,CA3,CA2,ANPEP,FPGS,CA4,KDR,CAPN1,MME,ITGA4,MMP1,PDE4D,IGFBP3,MMP2,PRKCD,PRKCA,AMPD3,MMP8,MMP9,MMP12,ACE2,ACLY,MMP14,FOLH1,MMP13,MMP16,PDE5A,PADI4,HPRT1,LAP3,RAF1,GART,ENPEP,HDAC2,PDE1B,HDAC1,GLO1,SLC5A2,HDAC6,PPP2CA,FYN,PCK1,EGLN1,ACE,ERAP1,BRAF,SELE,ESR1,SELP,NR4A1,WEE1,SELL,IMPDH2
GO:0016787	hydrolase activity	58	4.23E-23	5.93E-21	4.35	PRSS1,AMY2A,TREH,PTPN22,ECE1,CTSS,ADAMTS4,ADAMTS5,CASP7,CASP8,CASP6,CTSL,ANPEP,CASP3,CTSK,CASP1,CASP2,CTSH,CAPN1,CTSC,CTSB,MME,MMP1,PDE4D,MMP2,AMPD3,MMP8,F2,MMP9,BACE1,MMP12,ACE2,MMP14,FOLH1,MMP13,MMP16,PDE5A,PADI4,LAP3,MGLL,ENPEP,HDAC2,HDAC3,AHCY,PDE1B,HDAC1,HDAC6,PPP2CA,ELANE,CTSA,PTPN1,BCHE,MGAM,ACE,F10,CMA1,ERAP1,TMPRSS15
GO:0008233	peptidase activity	41	1.69E-29	7.10E-27	10.45	APP,PRSS1,ENPEP,ECE1,CTSS,ADAMTS4,ADAMTS5,CASP7,CASP8,CASP6,CTSL,CASP3,ANPEP,CTSK,CASP1,CASP2,CTSH,CAPN1,ELANE,CTSC,CTSB,CTSA,ACE,MME,F10,MMP1,CMA1,MMP2,ERAP1,MMP8,F2,MMP9,TMPRSS15,BACE1,MMP12,ACE2,MMP14,MMP13,FOLH1,MMP16,LAP3
GO:0000166	nucleotide binding	35	1.74E-07	4.88E-06	2.64	SRC,ADK,PYGM,PYGL,EGFR,PIK3CG,IGF1R,IKBKB,MAPK8,ERBB2,FPGS,KDR,AKT1,MAPK1,FYN,PCK1,SYK,CSNK2A1,INSR,PRKCD,CSNK2A2,DYRK1A,PRKCA,BRAF,MAPK14,ACLY,WEE1,LCK,IMPDH2,PDE5A,HPRT1,RAF1,MET,GART,MAP3K14
GO:0016740	transferase activity	35	4.40E-07	1.13E-05	2.54	SRC,GSTP1,ADK,PYGM,PYGL,TYMS,HDAC6,EGFR,PIK3CG,IGF1R,IKBKB,MAPK8,ERBB2,KDR,AKT1,MAPK1,FYN,PCK1,SYK,CSNK2A1,INSR,PRKCD,CSNK2A2,DYRK1A,PRKCA,BRAF,MAPK14,ACLY,WEE1,LCK,HPRT1,RAF1,MET,GART,MAP3K14
GO:0042802	identical protein binding	34	4.56E-07	1.13E-05	2.58	APP,PYGL,TNF,EGFR,PIK3CG,IGF1R,IKBKB,ADAMTS5,CASP8,TTR,CASP6,ERBB2,CASP1,KDR,CASP2,AKT1,MAPK1,CTSH,FYN,CTSC,BCHE,CSNK2A1,INSR,DYRK1A,BRAF,MMP9,ESR1,ACE2,NR4A1,LCK,PADI4,HPRT1,RAF1,MET

GO:0008270	zinc ion binding	34	3.67E-05	4.68E-04	2.11	KDM5B,ENPEP,THRB,THRA,GLO1,ECE1,HDAC6,ADAMTS4,ADAMTS5,CA1,CA3,CA2,ANPEP,CA4,EGLN1,PTPN1,ACE,MME,MMP1,MMP2,PRKCD,ERAP1,PRKCA,BRAF,MMP8,MMP9,ESR1,MMP12,ACE2,NR4A1,MMP14,MMP13,MMP16,RAF1
GO:0005524	ATP binding	30	1.35E-06	2.84E-05	2.69	SRC,ADK,PYGL,EGFR,PIK3CG,IGF1R,IKBKB,MAPK8,ERBB2,FPGS,KDR,AKT1,MAPK1,FYN,SYK,CSNK2A1,INSR,PRKCD,CSNK2A2,DYRK1A,PRKCA,BRAF,MAPK14,ACLY,WEE1,LCK,RAF1,MET,GART,MAP3K14
GO:0016301	kinase activity	27	1.08E-11	5.03E-10	5.14	SRC,ADK,EGFR,PIK3CG,IGF1R,IKBKB,MAPK8,ERBB2,KDR,AKT1,MAPK1,FYN,PCK1,SYK,CSNK2A1,INSR,PRKCD,CSNK2A2,DYRK1A,PRKCA,BRAF,MAPK14,WEE1,LCK,RAF1,MET,MAP3K14

Supplementary Table S4. Top 9 enriched Kyoto Encyclopedia of Genes and Genomes (KEGG) pathways.

KEGG Pathway ID	Pathway Name	Gene Count	p-value	Adjusted p-value (FDR)	Fold Enrichment	Genes
hsa01100	Metabolic pathways	39	1.14E-04	3.28E-04	1.84	AHCY,AMY2A,MAOB,PDE1B,GSTP1,TREH,GLO1,ADK,AKR1B1,PYGM,PYGL,HMGCR,TYMS,PIK3CG,CA1,ALDH2,CA3,CA2,ANPEP,FPGS,CA4,PCK1,MGAM,PDE4D,AKR1C3,AKR1C2,AMPD3,AKR1C4,DHODH,DHFR,ACLY,FOLH1,AKR1B10,IMPDH2,PDE5A,LAP3,HPRT1,GART,MGLL
hsa04010	MAPK signaling pathway	18	4.07E-07	2.80E-06	4.49	INSR,BRAF,PRKCA,MAPK14,TNF,EGFR,IGF1R,IKBKB,NR4A1,MAPK8,CASP3,ERBB2,KDR,AKT1,MAPK1,RAF1,MET,MAP3K14
hsa04151	PI3K-Akt signaling pathway	18	5.39E-06	2.43E-05	3.72	SYK,ITGA4,INSR,PRKCA,EGFR,IL2,PIK3CG,IGF1R,PPP2CA,IKBKB,NR4A1,ERBB2,KDR,AKT1,MAPK1,RAF1,PCK1,MET
hsa04668	TNF signaling pathway	13	5.01E-08	6.06E-07	8.17	MAPK14,SELE,TNF,MMP9,IKBKB,MMP14,CASP7,MAPK8,CASP8,CASP3,AKT1,MAPK1,MAP3K14
hsa04933	AGE-RAGE signaling pathway in diabetic complications	11	8.19E-07	5.11E-06	8.14	MAPK8,CASP3,MMP2,PRKCD,AGTR1,MAPK1,AKT1,PRKCA,MAPK14,SELE,TNF
hsa04068	FoxO signaling pathway	11	1.01E-05	4.25E-05	6.18	IKBKB,MAPK8,INSR,MAPK1,AKT1,BRAF,PCK1,RAF1,MAPK14,EGFR,IGF1R
hsa04910	Insulin signaling pathway	11	1.39E-05	5.37E-05	5.96	IKBKB,PTPN1,MAPK8,INSR,MAPK1,AKT1,BRAF,PYGM,PYGL,PCK1,RAF1
hsa04931	Insulin resistance	10	1.34E-05	5.37E-05	6.86	IKBKB,PTPN1,MAPK8,INSR,PRKCD,AKT1,PYGM,PYGL,PCK1,TNF
hsa04930	Type II diabetes mellitus	6	3.73E-04	8.87E-04	9.55	IKBKB,MAPK8,INSR,PRKCD,MAPK1,TNF
